# Mapping food surveillance chains through different sectors

**DOI:** 10.3389/fpubh.2023.1129851

**Published:** 2023-04-18

**Authors:** Laura Amato, Guido Benedetti, Paola Di Giuseppe, Viviane Hénaux, Renaud Lailler, Zuzana Nordeng, Tora Alexandra Ziesler Scharffenberg, Taran Skjerdal, Francesca Cito

**Affiliations:** ^1^Department of Epidemiology and Risk Analysis, Istituto Zooprofilattico Sperimentale dell’Abruzzo e del Molise, Teramo, Italy; ^2^Department of Infectious Disease Epidemiology and Prevention, Statens Serum Institut, Copenhagen, Denmark; ^3^Department of Communication, Istituto Zooprofilattico Sperimentale dell’Abruzzo e del Molise, Teramo, Italy; ^4^Laboratory of Lyon, Epidemiology and Support to Surveillance Unit, French Agency for Food, Environmental and Occupational Health and Safety (ANSES), Lyon, France; ^5^Laboratory for Food Safety, French Agency for Food, Environmental and Occupational Health and Safety (ANSES), Maisons-Alfort, France; ^6^Department of Research Administrative Support, Norwegian Institute of Public Health, Oslo, Norway; ^7^Department of Zoonotic, Food- and Waterborne Infections, Norwegian Institute of Public Health, Oslo, Norway; ^8^Department of Animal Health, Welfare and Food Safety, Norwegian Veterinary Institute, Ås, Norway

**Keywords:** One Health, surveillance, food safety, *Salmonella*, *Listeria*, Norway, France

## Abstract

European countries are investing in strengthening disease surveillance from a One Health (OH) perspective. During the MATRIX project, in the context of the One Health European Joint Programme, existing surveillance chains across the sectors of animal health, food safety, and public health have been investigated through questionnaires. Provided information has then been selected to be displayed in a single slide using an implemented mapping template. Two real-life scenarios are presented as case studies: the surveillance activities in place in France for *Salmonella* in the pork meat food chain, and in Norway for *Listeria monocytogenes* in the dairy food chain. The results collected through the questionnaires and the lessons learnt during the mapping process are reported, to share the advantages and drawbacks of the methodology. Moreover, the presented template could be adjusted and applied to different contexts. Mapping the components of existing disease surveillance systems is a fundamental step in understanding the relationships between its components, and subsequently facilitating their collaboration and integration under a OH approach.

## 1. Introduction

One Health (OH) defined as “an integrated, unifying approach that aims to sustainably balance and optimize the health of people, animals and ecosystems,” has become a widely accepted topic in the current debate about disease surveillance, and has a significant impact on the related health agenda (1–3). However, the practical application of the OH approach to real-life, existing surveillance systems is not easy. One Health surveillance (OHS) systems are not developed from scratch and the starting point is usually a combination of different hazard-specific problems, approaches, and objectives across the human, animal, and food safety sectors (4–6). Surveillance systems are complex structures and making the information gathered by a surveillance system useful for the involved stakeholders is not effortless (7–9). The OH approach necessarily adds complexity to existing surveillance systems and their chains of data flow. The complexity of the OH approach is related to the persistence of silo thinking (10), which, despite being effective and useful in terms of following up on specific actors and topics, complicates collaborations among actors within each segment of the ‘farm-to-fork’ chain.

European countries have invested in strengthening disease surveillance from a OH perspective with some successful collaborations, such as the Med.Vet.Net Association and the One Health European Joint Programme (OHEJP), which are now paving the way forward (11, 12). The OHEJP is a partnership between 44 European food, veterinary, and medical laboratories and institutes across Europe and the Med.Vet.Net Association (12). Among the many activities, including training opportunities and collaborations with the European intergovernmental agencies European Food Safety Authority (EFSA) and European Centre for Disease Prevention and Control (ECDC), the programme supports various research and integrative projects to stimulate the scientific development and integration of surveillance systems in a OH perspective (13).

In MATRIX, one of the OHEJP projects, the aim was to advance the implementation of OHS in practice, by building on existing resources, adding value to them, and creating synergies among sectors. The project created practical solutions for European countries to support and advance the implementation of OHS (14). MATRIX operated with a focus on specific pathogens/hazards (hazard tracks, HT) to ensure that the solutions developed by the project were relevant to their surveillance. The hazards were chosen in 2019, based on the operational priorities of the 19 MATRIX partner institutes across 12 European countries and their OH relevance, namely: *Campylobacter*, *Listeria monocytogenes, Salmonella*, and emerging threats, including Hepatitis E virus.

Prior to the integration of any surveillance system is the understanding of the relationships among its components. Mapping the components of existing disease surveillance systems is a fundamental step to facilitate subsequent integration of them from a OH perspective. As part of the broader objective to identify current examples of best practices and multi-sectorial collaborations across surveillance systems, one of the tasks of MATRIX aimed to map existing surveillance chains across the sectors involved in the surveillance of the project HTs, for at least one country per HT. Since the considered HTs are foodborne pathogens, the investigation followed the ‘farm-to-fork’ chain approach. The results of this work are detailed in a document published on Zenodo (15), the open repository developed under the European OpenAIRE programme. However, the mapping exercise allowed the identification of both opportunities and challenges of this investigation approach of what is already in place in different countries. In this paper, we therefore will describe our methodological approach, and be presenting two real-life scenarios as case studies.

The two scenarios chosen as case studies are the surveillance of *L. monocytogenes* in dairy products in Norway, and the *Salmonella* surveillance in pig meat in France. The scenarios concern pathogens that are of importance for human health based on the severity (*L. monocytogenes*) or the frequency (*Salmonella*) of the infections.

In 2020 listeriosis was the fifth most reported zoonosis (1,876 cases) in Europe, mainly affecting people over the age of 64 (16). In Norway, the number of annual cases of listeriosis in humans has been increasing gradually. Between 15 and 50 cases have been reported annually during the last decades, including a total of 37 cases in 2020 (17, 18). Given the severe symptoms and fatality rate of listeriosis cases, and a high probability of an increased human burden of disease, *L. monocytogenes* was ranked in the top five groups of biological hazards in a risk ranking and source attribution study carried out by the Norwegian Scientific Committee for Food and Health (19).

In general, the prevalence of *L. monocytogenes* in food is low, but the bacterium can grow rapidly when there are optimum conditions of pH, temperatures between 30 and 37°C, and a water activity of 0.99 (20). The theoretical minimum for growth is in conditions of pH 4.3, water activity of 0.92, and a temperature of −2°C, and both in presence or absence of oxygen (20). The minimum infectious dose is not known, but dose–response models indicate that the marginal probability of developing invasive listeriosis upon ingestion of one cell of *L. monocytogenes* per individual for the general population is 8 × 10^–12^, and 3 × 10^–9^ for extremely susceptible subpopulations (21). Applying this to concentrations of *L. monocytogenes* in food, these numbers fit with the observation that the estimated probability of illness increases at 1,000 cfu/g for the most vulnerable consumers and at 100,000 cfu/g for adults with no underlying illness, provided that the usual portion size is 100 g of food (22). When the growth conditions are good or the shelf life of the food is long, a high concentration of the bacterium can be reached before consumption. Foods with growth potential for *L. monocytogenes* that have a sufficiently long shelf life to exceed the critical concentrations mentioned above are regarded as risk products, unless they are heat-treated or *L. monocytogenes* is killed by other means before consumption. Contaminated, unpasteurised milk and other food ingredients are only some of the possible sources for the introduction of *L. monocytogenes* into dairies (23). *L. monocytogenes* can enter production facilities and remain for an extended time, even decades, contaminating the food at regular or irregular intervals (24). In addition, soft and semi-soft maturing cheeses are both examples of risk products for listeriosis. Outbreaks have been observed with cheeses from both pasteurised and unpasteurised milk: the largest in Norway was related to camembert cheese from a small-scale producer using pasteurised milk (25).

Dairy products are important both economically and culturally in Norway. Norwegian cheeses are, with only a few exceptions, produced and consumed domestically. In 2021, the annual consumption of cheese per person in Norway was 20,35 kg, of which 82% was produced in Norway (26). The import of cheese was about four times higher than the export (27). The variety of products from small-scale producers is large, and includes both pasteurised and unpasteurised products; the majority of dairy products sold are however coming from a few large producers, who produce from pasteurised milk and have extensive internal sampling programmes in place (15).

On the other hand, *Salmonella* is estimated to be responsible for more than 75 million foodborne infections worldwide each year (28). In Europe, salmonellosis was the second most frequent zoonotic disease reported, with more than 91,000 cases reported each year until 2018, representing an economic burden of around 3 billion euros (29). A marked improvement in this epidemiological situation can however be noted in comparison to the 200,000 annual number of human cases reported before 2004. The last Joint European zoonosis report from ECDC-EFSA highlighted decreasing number of human salmonellosis cases and *Salmonella* detection in food and animal sectors from 2016 to 2020. Nevertheless, this may be partly due to underreporting during the COVID-19 pandemic and Britain’s EU departure (16).

However, the number of positive sampling units related to the ‘pigs’ sector was stable in Europe over the same period (2016–2020). Pig meat and products thereof remained the second-largest source of salmonellosis food-borne outbreaks, with 11 strong-evidence outbreaks in 2020, compared to 37 outbreaks due to eggs and eggs products. Numerous *Salmonella* serovars were detected all along the food chain. Of these, *S.* Typhimurium, monophasic *S.* Typhimurium (1,4,[5],12:i:-) and *S.* Derby belonged to the top five, and were primarily related to pig sources (16). For these reasons above, the second scenario chosen as a case study is the *Salmonella* surveillance in pig meat in France.

In France, 139 among the 1,010 food-borne outbreaks declared in 2020 were attributable to *Salmonella* (120 were confirmed to have the presence of *Salmonella* in food, and 19 cases were suspected) (30). The annual number of illnesses attributable to *Salmonella* is estimated at 183,000, including 4,110 hospitalizations and 67 deaths (31). In France, 13 food-borne outbreaks were identified between 2002 and 2017, associated with products of porcine origin (32).

Contaminated raw animal food products are the main source of human infection. Contamination may occur during the processing stages from improper food handling and/or inadequate hygienic measures. Eating behaviours involving ingesting raw or undercooked products also pose a risk of infection (33). Most (42%) of reported cases of salmonellosis are linked to the consumption of eggs or egg products (34), but products from the pigs and dairy cattle sectors are also recognised as important reservoirs (35).

In pig farming, when an outbreak occurs, symptoms may include diarrhoea and growth delay. In farms with high biosecurity standards, the introduction of breeding animals and feed are considered the major routes for the introduction of *Salmonella*. Contamination of meat products most often occurs during the slaughtering of infected animals, when hygienic practices are lacking. For this reason, active monitoring is in place and is performed by the competent authority. In 2020, French food business operators (FBOs) performed more than 14,000 official controls at slaughterhouses and detected 4.8% (IC 95%: [4.4–5.2]) of pig carcasses contaminated by *Salmonella* (16).

At this stage, however, the integrated surveillance of *Salmonella* in the pig sector does remain needed in France. A shift towards a multi-sectorial approach is currently ongoing with the implementation of a collaborative and multidisciplinary platform dedicated to food chain surveillance (36).

The purpose of the paper is to describe the methodological approach we used to map the components of the existing disease surveillance systems for these two case scenarios, to enable its further application, and to share the lesson learned.

## 2. Materials and methods

### 2.1. Online questionnaires

Within the activities of the project MATRIX, a multiple-choice questionnaire was created for each of the four hazards (*Salmonella, Campylobacter, L. monocytogenes*, and Hepatitis E virus), to gather the necessary information for the mapping of the existing food chain surveillance activities from national experts in the field. As an adaptation of the approach from ‘farm-to-fork’ to ‘farm-to-patient’, each questionnaire was divided into three different sections: (I) focusing on the animal health aspects (AH), (II) on the food safety aspects (FS), and (III) on public health (PH). In each section, the surveillance was assessed by gathering information on actors, sampling context, collected sample types, laboratory methods for diagnosis, available data sources, and cross-sectoral collaboration in place. To ensure to include all the relevant information, eight experts were consulted during the implementation of the specific questionnaires for each sector.

The draft version was circulated amongst the MATRIX participants for evaluation and implementation. The MATRIX partners were asked to suggest possible contact persons with expertise in the specific field of interest, between project partners and non-partners institutions. The identified experts were individually contacted to verify their interest and availability in taking part in the survey. The final version of the questionnaires was put online on the survey platform Survey Monkey©, for dissemination to the relevant experts previously selected. Given the specificities of the information required, a PDF version of the questionnaires (see Supplementary material, modified with permission from Cito et al., 2022 (15)).

### 2.2. Mapping template

A questionnaire was considered completed when answers from the three involved sectors (AH, FS, PH) were obtained. Upon the reception of the three compiled sections, a preliminary evaluation of the results was carried out. Where missing or unclear information emerged, we requested clarifications by re-sending the questionnaire to the reference expert (or to a different one). For this reason, the questionnaires were open for completion for a period of about six months.

In order to evaluate and display the collected information, a categorisation was put in place: information was classified as part of ‘data’, ‘metadata’, ‘events’, ‘event producing data (EPD)’, and/or ‘identified data source (IDS)’ (15).

**Figure 1:**
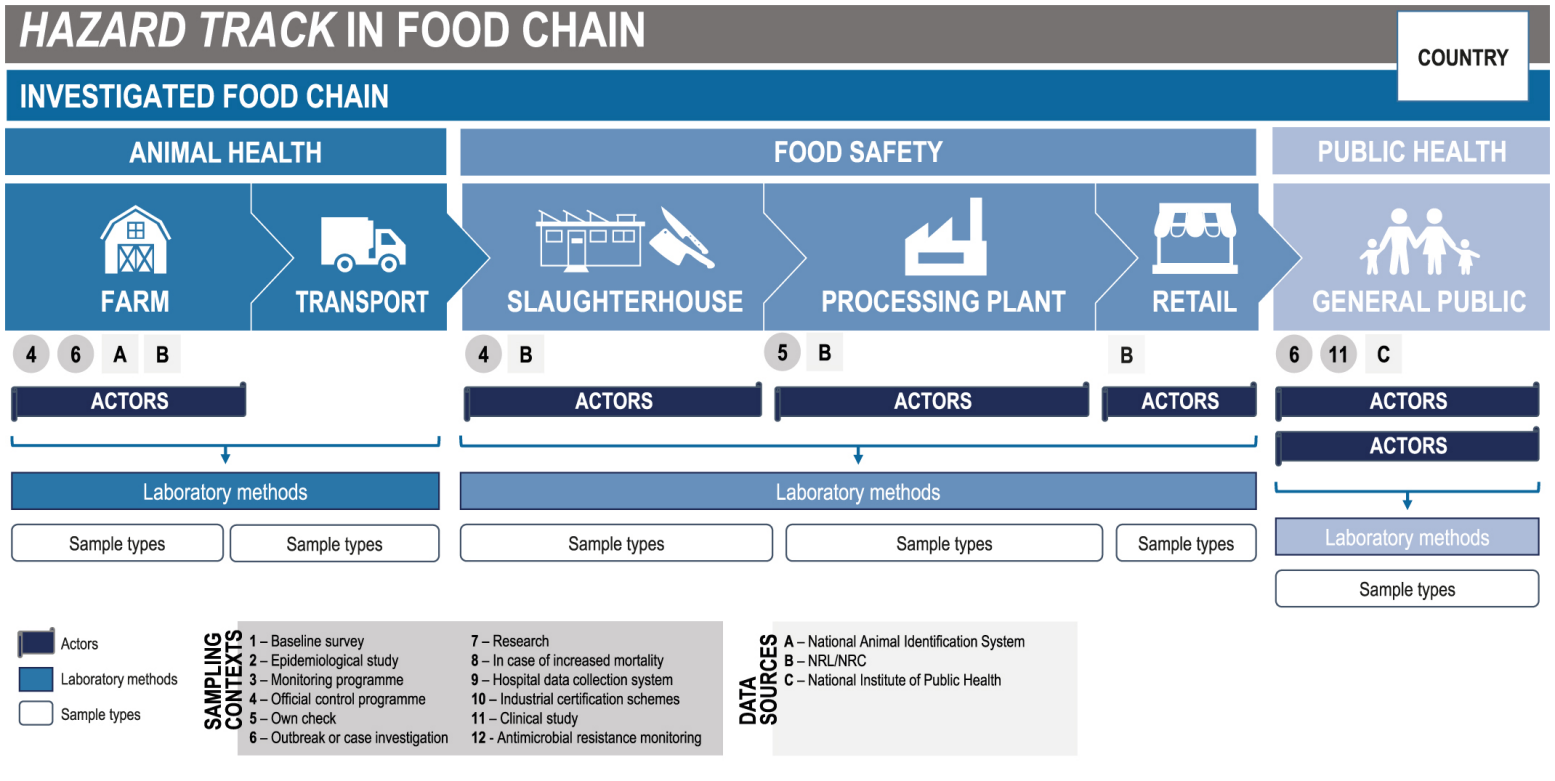
Mapping template. Modified with permission from Cito et al., 2022 (15).

The subsequent step was then the identification of the most relevant information, for their graphic representation on a map. Therefore, the information regarding the actors, the sampling context, the collected sample types, the laboratory methods in use in the diagnosis, and the available data sources, for each one of the sections, were highlighted. For the purpose of the task, we designed a template of the mapping and displayed it using MS PowerPoint^©^ (Figure 1).

### 2.3. The two case studies

One of the main objectives of the MATRIX project was to map the surveillance systems along the food chain. To achieve this objective, we selected a specific food chain to be investigated in detail per each hazard. Combinations that are relevant from the public health point of view were selected, based on a consensus among the MATRIX Consortium on the epidemiological situation in 2020 in Europe.

Concerning *Listeria*, the selected food chain was dairy products, given the epidemiological relevance of these products for the transmission of *L. monocytogenes* to humans. The investigated country was Norway, because of the economic and cultural importance of dairy products (23, 37).

Regarding *Salmonella*, we decided to assess surveillance activities in France in the pork meat food chain to avoid overlapping with the OHEJP project NOVA (38), which investigated the poultry food chain with regard to *Salmonella* surveillance activities. For this reason, some information was already available, while less information existed for the pork meat food chain and the same pathogen.

## 3. Results

We present below the results collected through the questionnaires on *L. monocytogenes* in dairy products in Norway, and *Salmonella* in the pork meat food chain in France, based on the information provided by the experts involved.

### 3.1. Listeria

**Figure 2:**
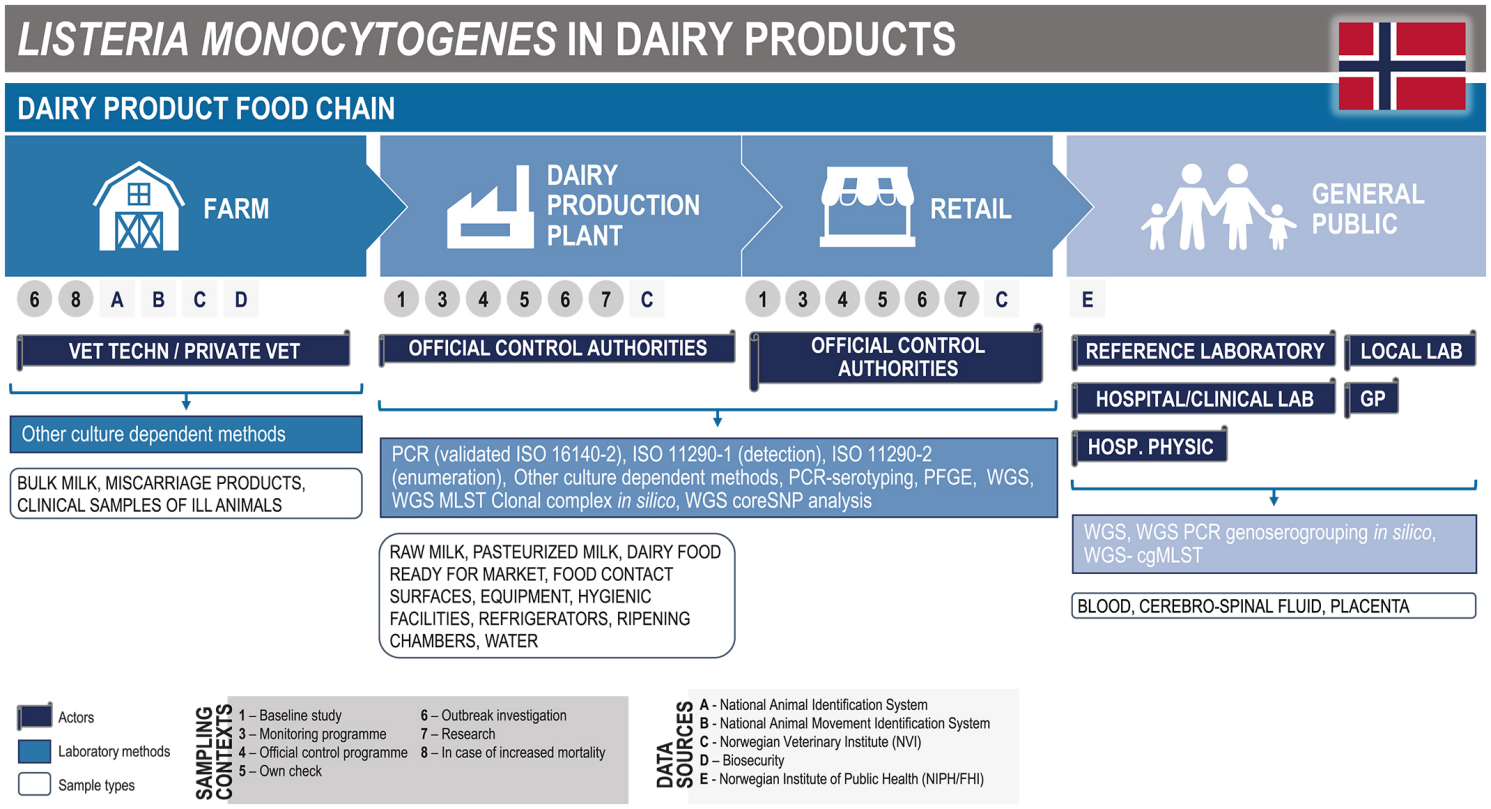
*Listeria monocytogenes* mapping. Modified with permission from Cito et al., 2022 (15).

In Norway, the national and regional surveillance programmes in place are designed to detect illness cases among humans and animals, and non-compliance to food safety criteria in food, adapted to different production routes (Figure 2).

#### 3.1.1. Animals

Veterinary technicians and/or private veterinarians carry out surveillance activities in the animal sector and perform outbreak investigations in case of increased mortality. Abortions are investigated, and bulk milk and blood from sick animals are collected. The bulk milk is routinely analysed at large-scale dairies, where the focus is on milk quality and production hygiene indicators rather than on *L. monocytogenes* specifically. Neurolisteriosis (meningitis) in animals is not a notifiable disease in Norway: clinical cases are not registered systematically, and clinical suspects are only rarely confirmed by laboratory diagnosis. The few laboratories that are involved in the diagnostics of listeriosis in animals work collaboratively at the national level. Even though laboratory results are not shared automatically, information can be made available upon request. The number of confirmed animal cases per region is reported and shared at the national level (15).

#### 3.1.2. Foods

The sampling plans in the official national programmes are designed to cover imported foods and local small-scale dairy products. Large-scale dairies usually have their own sampling programmes. The surveillance of small-scale producers includes the sampling of summer products. In some programmes, ‘24 h samples’ (which means sampling the day after the start of the maturation process) are implemented in farms and small-scale dairies, as several pathogens can be found at the highest concentration at this stage. This kind of sampling allows for the rapid detection of anomalies and allows for sampling without the loss of the entire cheese.

Sampling is also performed at the retail level, in compliance with the microbial criteria in the food legislation. In addition, metadata like production date, shelf-life date, animal species, whether the product is made of pasteurised or unpasteurised milk, producer, sampling place (address and kind of shop), and sampler can be recorded. For all products, a picture of the product is also collected. Auditors from the official control authorities carry out the sampling and the follow-up of positive samples with the producers.

The National Reference Laboratory for *Listeria* in food, which is represented by the Norwegian Veterinary Institute (NVI), carries out the analysis of *L. monocytogenes* and other microbes. Detection and enumeration of *L. monocytogenes* are always included in the analyses. Whole genome sequencing (WGS) is newly applied, while it was not fully operational at the time at which the questionnaire was available for response. Isolates are stored for further analyses, for instance in case of outbreak investigation or research. Positive results are directly notified to the auditors, to allow rapid outbreak investigations and direct follow-up in case of non-compliance. In addition, all the results are anonymised, categorised, and presented annually or at the end of the programme. However, the national active surveillance programme for cheese and milk products is adapted intermittently: the focus foods for surveillance are decided every 1–3 years, based on priority lists for hazards and foods of particular concern.

Besides the official surveillance programme, the farmers and dairies have their own-check sampling programmes in place, and hazard analysis and critical control points (HACCP) plans. Sampling in these cases may include the testing of surfaces, equipment, refrigerators, and water.

#### 3.1.3. Humans

Human listeriosis in Norway has been nominatively notifiable in the Norwegian Surveillance System for Communicable Diseases (MSIS) (39) since 1991 (NIPH, 2022). Age, gender, place of residence, and travel history are among the parameters collected. The official number of cases is updated daily (15).

Medical microbiological laboratories in Norway are obligated to send clinical *L. monocytogenes* isolates to the National Reference Laboratory for Enteropathogenic Bacteria at the Norwegian Institute for Public Health (NIPH). WGS is performed routinely for confirmation, surveillance, and outbreak purposes (NIPH, 2022). All listeriosis cases are routinely investigated with a trawling questionnaire. When a WGS cluster is detected, epidemiological parameters as well as information from the trawling questionnaire are considered before the outbreak investigation is initiated.

During an outbreak investigation, the NIPH works in close collaboration with municipality doctors, the Norwegian Food Safety Authority, and the NVI.

### 3.2. Salmonella

In France, the *Salmonella* surveillance is based on a national system composed of approximately fifteen components or networks (36). The system covers the entire food chain and most populations who are more at risk for these pathogens. Surveillance aims at reducing the risk for consumers through earlier detection of contamination by *Salmonella* in the food chain, limiting the economic impact of these contaminations in the production chains, and advancing knowledge.

**Figure 3:**
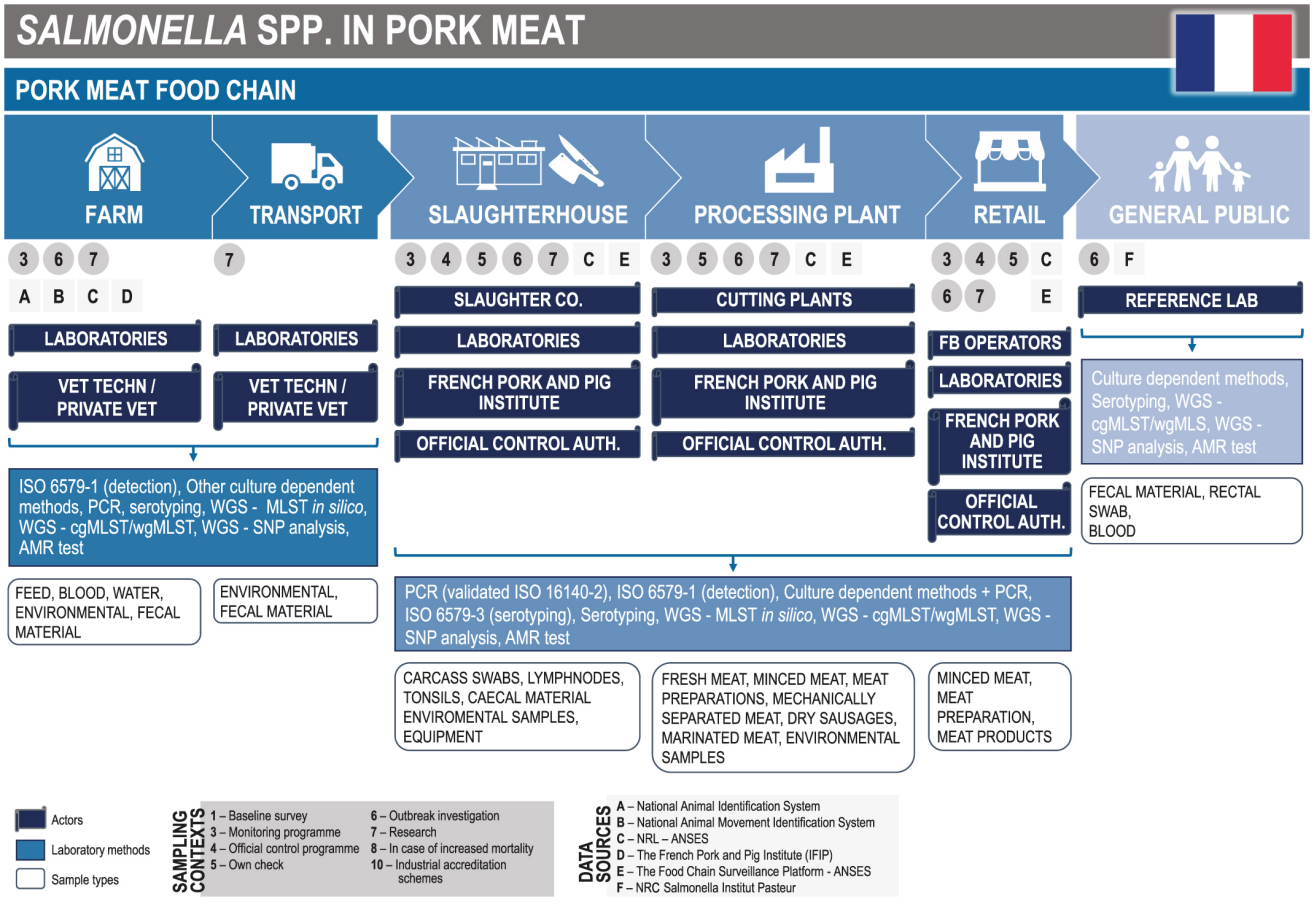
*Salmonella* mapping. Modified with permission from Cito et al., 2022 (15).

The French Public Health Institute, named ‘Santé publique France’ (SpF), defines a foodborne outbreak at the national level as the occurrence of at least two cases of similar symptomatology, generally gastrointestinal, which are attributed to the same food origin. The notification of cases has been mandatory since 1987. A notification can lead to investigations through the whole food chain and within different animal and food production sectors (Figure 3). In the past, the pork food chain has been impacted on several occasions by *Salmonella* contamination (40, 41).

#### 3.2.1. Animals

In the animal sector, many activities for *Salmonella* surveillance are implemented at the farm level in France (Figure 3), which are carried out by official control authorities, laboratories, farmers, the industry, private veterinarians or technicians, and eventually research centers or institutions like universities.

In the framework of monitoring programmes, outbreak investigations, or research projects, these actors collect environmental samples, including fecal material, water, and feed to detect and identify the bacteria by phenotypic or molecular methods. Laboratories implement official methods to serotype all isolates and, among this panel, only a part of the samples is typed in depth by polymerase chain reaction (PCR), SNPs, or cgMLST. All strains isolated in an outbreak context are sequenced with the technical support of the National Reference Laboratory (represented by the French Agency for Food, Environmental and Occupational Health and Safety - ANSES). These surveillance activities (through research) also concern animal movements.

The monitoring and control of the application of biosecurity measures are particularly important, for both breeding and fattening pig farms. For this reason, additional data including personnel movement, and records of cleaning and sanitation procedures, is collected. The French Pork and Pig Institute (IFIP) stores the collected data at national and regional levels, and shares with other actors information on the coverage of surveillance activities and descriptive epidemiological results.

#### 3.2.2. Foods

For the food sector, official control authorities, the private sector, laboratories, and the IFIP predominantly perform activities at the slaughter and processing plants. Carcass swabs sampled at the slaughterhouses for official control programmes, are collected with other samples retrieved from the environment and equipment during monitoring programmes, own-checks, or outbreak investigations. Information on the activities performed at the retail stage, provided through the questionnaires, included that minced meat and meat preparations/products are subject to monitoring and research activities, outbreak investigations, official control programmes, and own-check.

In France, sampling conducted within established surveillance programmes aims to investigate the exposure to *Salmonella* spp. In addition, sampling is targeted at consumer groups (e.g., vulnerable consumers, and consumers of a high amount of a particular food), and import/export. In case of non-compliance, depending on the results of the risk analysis, additional analyses may be carried out on the relevant products. Routinely, laboratories test samples for *Salmonella* detection by culture-dependent and molecular methods based on PCR. Each isolate is serotyped by the method of reference (ISO 6579-3:2017). WGS is performed to type strains that are suspected to be linked to food-borne outbreaks when epidemiological evidence (descriptive or analytical) is limited. The percentage of typed strains depends on the context but represents only a small fraction of the isolated strains. The overall process of testing and reporting may take months to conclude, even if the testing process is typically quite rapid.

In 2018, the Food Chain Surveillance Platform was created to support surveillance activities and to promote an operational OH approach at the national level. This innovative structure is based on public and private governance. It effectively coordinates notably working groups on *Salmonella* with stakeholders including the IFIP, the *Salmonella* National Reference Laboratory (NRL), and National Reference Center (NRC), which are both hosted by a research unit from ANSES and Institut Pasteur, respectively, and numerous partners involved in the French *Salmonella* surveillance system (36).

#### 3.2.3. Humans

In France, sporadic cases of salmonellosis are not notifiable diseases. Several actors, from local health authorities to hospitals/clinical/reference/local laboratories, monitor for human salmonellosis. In general, consistent data related to case detection are collected on a routine basis, while additional epidemiological data are collected mainly during outbreak investigations.

A research unit from Institut Pasteur hosts the French mandate of NRC for *Salmonella*. This reference laboratory collects strains and data related to human cases confirmed by contaminated blood or faecal material. NRC shares confidential data related to each case with SpF, including the severity of symptoms, and spatial and temporal data. WGS is systematically performed, and results are centralised. Algorithms using this database produce weekly alerts when clusters based on microbiological data occur, and then the NRC informs SpF of these situations. Currently, there is no automatic tool or shared database in place at the national level to allow prompt interaction between human and non-human sectors. To date, the ability to share data mainly depends on the interpersonal connections between scientists working at the reference laboratories (NRC and NRL).

In conclusion, the collaboration between sectors exists mostly for foodborne outbreak surveillance and investigation. The exchange of information issued from investigation frameworks is in place between the Regional sanitary authorities in charge of human surveillance (‘Regional health agency’) and of food safety, animal health, and welfare (‘Departmental Directorate for Social Cohesion and Population Protection’). Additionally, information is shared with the national competent authorities to implement adjusted control measures. The NRC and NRL have a central position in the framework, managing laboratory networking, developing, and harmonising analytical methods, and interacting with administrative organisations and professional and technical centers (including research).

## 4. Discussion

### 4.1. The online questionnaires

The methodological approach adopted during the MATRIX project included the use of online questionnaires to collect information about surveillance in place in European countries. Our approach allowed for a substantial set of information to be obtained, in terms of both quality and quantity.

Although in some cases surveillance activities are regulated by the existing European legislation [i.e., control programmes regarding *Salmonella* (42), official controls under Regulation 2017/625 (43) to verify that food complies with microbiological and process hygiene criteria established by Regulation 2073/2005 (44) or epidemiological surveillance of communicable diseases (45)], in other there is no harmonised surveillance in the European Union. For this reason, the collection of information from the existing European legislation would have represented only a fraction of the overall amount of information gathered by the questionnaires.

The questionnaires mainly asked closed questions with multiple-choice answers and checkboxes. This can potentially lead to biases, defined as a ‘deviation of results or inferences from the truth, or processes leading to such a deviation’ (46). The biases may particularly result from the design of the questions and questionnaires, and/or from their modalities of administration and completion (46). Semi-directive interviews may have allowed for collecting information that is more comprehensive. However, the conduction of interviews would have been more time-consuming and potentially introduced a greater risk of biases, given the interviewer’s subjectivity. Moreover, the use of questionnaires was a good alternative to in-person workshops, which were not feasible during the period of travel restrictions due to the SARS-CoV-2 pandemic. The questionnaire and the subsequent mapping made possible the drawing up of the initial description of the surveillance structure as the starting point for working collectively, and in more detail on each aspect.

When using online questionnaires to collect information, the implementation can be an involved process, and it requires resources with expertise to design, pilot, and put them online. Both compiling and responding to the questionnaires also require deep knowledge of the subject. Therefore, depending on the involved expert in the compilation and response respectively, possible biases may be introduced. In addition, the splitting of the questions according to the three investigated sectors could not be sufficient, because even within the same sector the skills are diversified. As consequence, it could not be expected that each expert had the expertise to cover all aspects included in a single sector questionnaire (i.e., from the surveillance programmes in place, to existing information systems, and to laboratory tests used for diagnosis).

To mitigate these risks, we applied the approach of involving, first, a country expert within the OHEJP MATRIX partner institutes and asking them to share the questionnaires with the appropriate experts, which could belong to different agencies. In this way, we gathered information not only from project partners but also from all three sectors involved in the surveillance of the pathogen under investigation.

### 4.2. Mapping template

The mapping process could be a key step in initiating collaborative work to set up or improve a surveillance system. It seemed essential to clearly identify the actors involved in the monitoring, their role, and their position in the organisation, before considering implementation or possible adaptations and changes as actions, to achieve pre-established consensual objectives (36).

Although some examples of mapping were already available (47), we designed a new template to display the relevant actors and other data regarding HT-specific surveillance. The key aspect of the mapping is the presentation, with a single figure, of the three investigated sectors, and for each sector the implemented surveillance activities. In this way, a clear visualisation and a quick comparison of the information reported is possible and the One Health approach is represented.

The three involved sectors were animal health, food safety, and public health. Besides the food safety area, the OH approach can be applied to many others, covering complex health issues and requiring close collaboration across sectors, stakeholders, and countries (48). Hence, our template can be applied to several different contexts, by simply adjusting the underlying structure. Beyond the purpose of the MATRIX project, in which a method to display/map surveillance activities was developed, the same method could be applied to several other scenarios. As a generic approach, the implementation of this template could facilitate also the description of areas within chemical monitoring, for example, using a preliminary adaptation of the questionnaire. Across further applications, the mapping approach could cover a whole production sector, impacted by several contaminants, or a specific contaminant monitored by multiple production sectors.

### 4.3. The two case studies

In this study, we emphasised the methodology rather than the data collected using the questionnaire. Significantly more data than those shown on the maps were collected. The complete results are enclosed in a specific deliverable of the MATRIX project (15). Here, we presented the application of the mapping of *L. monocytogenes* in Norway and *Salmonella* in France, as they were representative of two situations in which such information was thoroughly reported.

The discussion with the experts on the two case studies highlighted how communication between official partners is generally more efficient when colleagues from different sectors know each other. Direct familiarity and trust can be important added values for successful surveillance and outbreak investigations (49).

The mapping clearly showed that surveillance of the animal and food sectors needs to be specifically designed to catch the production, processing, and use of the food products, by covering features such as seasonality, regional differences within a country, and large versus small-scale productions. The mapping method could be particularly useful in the case of a food category with a domestic market and small-scale producers, to follow up with the producers who do not have the size or economy to carry out many analyses. The additional value of using this approach, besides building connections and trust among authorities and producers, is to identify conditions that could lead to outbreaks, rather than detecting outbreaks when they have already started. The approach of having sampling schemes designed for the detection of risk factors within each sector, and combined with suited characterisation analyses and data sharing with other relevant sectors, can result in cost savings and rapid detection of OH challenges, regardless of the original purpose of the surveillance programme.

For the food health segment, the focus has been placed on the consumers. It is possible to arrange different surveillance programmes for various vulnerable groups, but this aspect is already targeted in passive surveillance systems, when consumers go to the doctor if they are ill. The human health surveillance programme operates in a similar manner, regardless of the food segment covered. The contact between animal, food, and the human sector is likely to be easier for domestically produced and consumed food, as the options for signaling are more between people who know each other and work together on a regular basis, than if animal, food, and human health segments need to be alerted with official channels first.

However, it is critical to define the specific situations under which other sectors should be alerted, and what information (in terms of data and metadata) should be shared among the different identified actors. Generally, the implementation of the OH approach is easier under the circumstance of an outbreak, since all the involved actors have the common goal of identifying the source of the infection and implementing control measures. The same thing does not happen during routine surveillance. Therefore, there is a general need for ‘traffic lights’ and checkpoints, about what to share, when, and why. While it is true that trust is important for sharing and respecting the rules agreed upon, active communication between sectors is a prerequisite for building trust. Collaborations are established gradually, based on the adhesion of the partners to a common organisation. A mapping stage could therefore be a prerequisite for establishing a shared and integrative vision of the organisation of surveillance activities, as a ground for further collaborative efforts. As an example, an approach to OH surveillance of listeriosis was suggested already in 2001 from France but was not followed up by other countries (50). The current work in France and Norway to improve the efficiency of food hazard surveillance throughout the food chain is highlighting how long, sensitive, but successful, the process is.

However, these food-borne hazards are not solely present within specific countries but are widespread in Europe and beyond. Because animals, food, and people move between countries, establishing links between specific country hazard maps would be useful. Likewise, efforts towards a OHS should be first made at the national level, and at some point linked internationally.

## 5. Conclusion

During the MATRIX project, we proved that it is possible to map surveillance chains of foodborne pathogens of One Health relevance across the human health, animal health, and food safety sectors in various European countries, and the methodological approach described in this manuscript is replicable in several contexts. Although many efforts are implemented to remove barriers to a better application of the One Health, the importance of shifting from silo thinking should not be underestimated. The methodological approach that we presented can support identifying new opportunities for integrating OHS, while lifting our heads and looking further than we normally do, as it happens during research projects.

## Data availability statement

The raw data supporting the conclusions of this article will be made available by the authors upon request, without undue reservation.

## Ethics statement

Ethical review and approval was not required for the study on human participants in accordance with the local legislation and institutional requirements. Written informed consent from the participants was not required to participate in this study in accordance with the national legislation and the institutional requirements.

## Author contributions

LA, GB, PG, TSk, and FC: idea and conceptualization. LA, FC, and TSk: methodology. LA, PG, and FC: data curation. LA, GB, VH, RL, ZN, TSc, TSk, and FC: original draft preparation and revision and editing. FC: supervision. All authors contributed to the article and approved the submitted version.

## Funding

This work was supported by funding from the European Union’s Horizon 2020 Research and Innovation Programme under grant agreement no. 773830: One Health European Joint Programme.

## Conflict of interest

The authors declare that the research was conducted in the absence of any commercial or financial relationships that could be construed as a potential conflict of interest.

## Publisher’s note

All claims expressed in this article are solely those of the authors and do not necessarily represent those of their affiliated organizations, or those of the publisher, the editors and the reviewers. Any product that may be evaluated in this article, or claim that may be made by its manufacturer, is not guaranteed or endorsed by the publisher.

## Supplementary material

The Supplementary material for this article have been modified with permission from Cito et al., 2022 (15), and can be found online at: https://www.frontiersin.org/articles/10.3389/fpubh.2023.1129851/full#supplementary-material
