# Supplementary material for: Mapping food surveillance chains through different sectors
Source: Front Public Health. 2023 Apr 18;11:1129851. doi: 10.3389/fpubh.2023.1129851 (PMC10151742; doi:10.3389/fpubh.2023.1129851)
Supplement: Supplementary file 1 [file Data_Sheet_1.ZIP › LISTERIA - SECTION B.pdf]

LISTERIA IN HUMANS AND IN DAIRY PRODUCTS FOOD CHAIN - SECTION B

SURVEILLANCE OF DISEASE IN ANIMALS

Dear participant,
in the context of the OHEJP Matrix, Work-Package 2 (Best-practices and multi-sectorial collaboration) was implemented this online questionnaire to collect information about the surveillance of **Listeria** in the **dairy products food chain**, in three sectors: public health, animal health, and food safety.

We would like to thank you for your willingness to fill in the questionnaire for the **animal health** sector.

Please find hereby some information regarding personal data processing.

Under Articles 13 and 14 of Regulation (EU) 2016/679, the personal data processing concerns the personal data - name, family name, institution, email address - of those who answer the questionnaire as part of the Matrix project.
The data controller is the legal representative of Istituto Zooprofilattico Sperimentale Abruzzo e Molise "G. Caporale" – Teramo (Italy), www.izs.it - protocollo@pec.izs.it, +3908613321. The contact details of the DPO of the Institute are: dpo@izs.it, dpo@pec.izs.it, +39 0861 3321.
Personal data collected will be processed for purposes connected with the handling of the contractual requirements related to the management of the Matrix project (art. 6, § 1, letter b) of Regulation).
The personal data provided will not be subject to communication and/or dissemination.
All personal data collected will be processed electronically on digital medium using the specific information systems and, in any case, the processing is made exclusively by personnel in charge. All data collected will be retained anonymously on digital.
At any time, data subjects have the right to ask the data controller for accessing their personal data, confirming such data exist, to know the content, the origin, and the processing terms, to request the update, the rectification, the erasure, the transformation into anonymity or the blocking of the data processed in breach of the law or to object the processing. The related request should be made by contacting the Data Protection Officer or the supervisory authority, in particular in the Member State of his or her habitual residence.

* 1. Country

* 2. Contact info of the person replying to the questionnaire

Name and Surname

Institution

E-mail address

LISTERIA IN HUMANS AND IN DAIRY PRODUCTS FOOD CHAIN - SECTION B

SURVEILLANCE

3. Is Listeriosis in milk-producing animals (e.g. cows, sheep, and goats) a notifiable disease* in your country?

** "A disease that, by law, must be reported to public health authorities upon diagnosis." (EJP ORION Glossary)*

- ◯ YES
- ◯ NO

4. Is there any legal (official) “case definition” and/or “outbreak definition” in your country?

- ◯ YES
- ◯ NO

5. If yes, please specify the “case definition” and/or “outbreak definition”

6. Is the surveillance* for *Listeria* spp. in milk-producing animals in place in your country?

**Surveillance understood as “Targeted Surveillance that is focused on one or more pre-defined hazards (disease, condition, biological, chemical or physical agent, or event) often this form of surveillance uses diagnostic tests for the detection of particular pathogens (e.g. molecular diagnostic methods).” (EJP ORION Glossary)*

- ◯ YES
- ◯ NO

7. Are different types of surveillance activities in place for *Listeria* spp. in milk-producing animals carrying out based on:

	EU legal obligation	National legal obligation	Voluntary based
Official control program	☐	☐	☐
Baseline survey	☐	☐	☐
Monitoring program	☐	☐	☐
Research project	☐	☐	☐
Outbreak investigation	☐	☐	☐
Other	☐	☐	☐

Other (please specify)

8. Who are the actors in charge of carrying out surveillance activities for *Listeria* spp. in milk-producing animals?

- ☐ Farmer
- ☐ Official Control Authorities
- ☐ Vet technician/Private Vet
- ☐ Other (please specify)

9. How often are the samplings performed in milk-producing animals?

	Ongoing	Monthly	Quarterly	As required *(E.g. as a part of a defined study or during a defined period)*
Official control program	☐	☐	☐	☐
Baseline survey	☐	☐	☐	☐
Monitoring program	☐	☐	☐	☐
Research project	☐	☐	☐	☐
Outbreak investigation	☐	☐	☐	☐
Other	☐	☐	☐	☐

Other (please specify)

10. If the data collection is “ongoing”, what is the required notification time period?

- ◯ 24 hours
- ◯ 48 hours
- ◯ one week
- ◯ two weeks
- ◯ Other (please specify)

11. What types of specimens are collected during surveillance activities for *Listeria* spp. in milk-producing animals?

	Official control program	Baseline survey	Monitoring program	Research project	Outbreak investigation
Individual milk	☐	☐	☐	☐	☐
Bulk milk	☐	☐	☐	☐	☐
Fecal material	☐	☐	☐	☐	☐
Miscarriage products	☐	☐	☐	☐	☐
Environmental surfaces sampling	☐	☐	☐	☐	☐
Feed	☐	☐	☐	☐	☐
Water	☐	☐	☐	☐	☐
Other	☐	☐	☐	☐	☐

Other (please specify)

12. Is surveillance* for *Listeria* spp. in milk-producing animals in place in case of increased mortality?

**Surveillance understood as “The real-time (or near real-time) collection, analysis, interpretation and dissemination of health-related data to enable the early identification of the impact (or absence of impact) of potential human or veterinary public health threats which require effective public health action”. (EJP ORION Glossary)*

- ◯ Yes
- ◯ No

13. In case of yes, which organs/tissues of the animal are investigated?

LISTERIA IN HUMANS AND IN DAIRY PRODUCTS FOOD CHAIN - SECTION B

DATA MANAGEMENT AND LABORATORY METHODS

14. Do you have a National Identification System of milk-producing animals in place in your country?

- ◯ Yes
- ◯ No

15. Do you have a National Animal Movement Identification System of milk producing animals in place in your country?

- ◯ Yes
- ◯ No

16. Are data on surveillance activities in place for *Listeria* spp. in milk producing animals stored in electronic data collection systems at national level?

- ◯ Yes
- ◯ No

17. Please, provide the name and contact details of the institution in charge of collecting and storing data coming from surveillance activities at the national level.

Institution	
City	
Website	

18. What kind of information collected during surveillance is shared at national level?

- ☐ Number of confirmed cases
- ☐ Number of suspected cases
- ☐ Number of depopulated animals
- ☐ Number of dead animals
- ☐ Type of specimen *(bulk milk, miscarriage products, etc.)*
- ☐ Sampler
- ☐ Date of sample collection
- ☐ Place of sample collection
- ☐ Sampling context *(official control program, monitoring, etc.)*
- ☐ Other (please specify)

19. Please specify the laboratory test used routinely for each type of specimen

	Individual milk	Bulk milk	Fecal material	Miscarriage products	Environmental surfaces sampling	Feed	Water
PCR	☐	☐	☐	☐	☐	☐	☐
ISO 11290-1 (detection)	☐	☐	☐	☐	☐	☐	☐
ISO 11290-2 (enumeration)	☐	☐	☐	☐	☐	☐	☐
Other culture dependent methods	☐	☐	☐	☐	☐	☐	☐
Culture dependent methods + PCR	☐	☐	☐	☐	☐	☐	☐
Other	☐	☐	☐	☐	☐	☐	☐

Other (please specify)

____

20. Are the Listeria strains characterized?

- ◯ Yes
- ◯ No

21. If yes, which laboratory methods are used routinely to characterize Listeria strains?

	Always	Sometimes	Never
Serotyping	◯	◯	◯
PCR serotyping	◯	◯	◯
MLST	◯	◯	◯
PFGE	◯	◯	◯
Whole genome sequencing	◯	◯	◯
WGS - PCR serotyping in silico	◯	◯	◯
WGS - MLST in silico	◯	◯	◯
WGS - Clonal Complex in silico	◯	◯	◯
WGS - cgMLST	◯	◯	◯
WGS - wgMLST	◯	◯	◯
WGS - coreSNP analysis	◯	◯	◯
WGS - wgSNP analysis	◯	◯	◯
Antibiotic susceptibility testing	◯	◯	◯
Other	◯	◯	◯

Other (please specify)

22. How do you share the results of laboratory methods? Please check for each diagnostic test one or more possibilities

	National level	Sub-national / Regional level	Local level	Intersectorial: human, animal, food	Not shared
PCR	☐	☐	☐	☐	☐
Culture dependent methods	☐	☐	☐	☐	☐
Culture dependent methods + PCR	☐	☐	☐	☐	☐
Enumeration	☐	☐	☐	☐	☐
Serotyping	☐	☐	☐	☐	☐
PCR serotyping	☐	☐	☐	☐	☐
MLST	☐	☐	☐	☐	☐
PFGE	☐	☐	☐	☐	☐
Whole genome sequencing	☐	☐	☐	☐	☐
WGS - PCR serotyping in silico	☐	☐	☐	☐	☐
WGS - MLST in silico	☐	☐	☐	☐	☐
WGS - Clonal Complex in silico	☐	☐	☐	☐	☐
WGS - cgMLST	☐	☐	☐	☐	☐
WGS - wgMLST	☐	☐	☐	☐	☐
WGS - coreSNP analysis	☐	☐	☐	☐	☐
WGS - wgSNP analysis	☐	☐	☐	☐	☐
Antibiotic susceptibility testing	☐	☐	☐	☐	☐
Other	☐	☐	☐	☐	☐

Other (please specify)

23. When are the results shared for each analytical method used?

	Routinely	Outbreak investigation	Research	As required *(E.g. as a part of a defined study or during a defined period)*
PCR	☐	☐	☐	☐
Culture dependent methods	☐	☐	☐	☐
Culture dependent methods + PCR	☐	☐	☐	☐
Enumeration	☐	☐	☐	☐
Serotyping	☐	☐	☐	☐
PCR serotyping	☐	☐	☐	☐
MLST	☐	☐	☐	☐
PFGE	☐	☐	☐	☐
Whole genome sequencing	☐	☐	☐	☐
WGS - PCR serotyping in silico	☐	☐	☐	☐
WGS - MLST in silico	☐	☐	☐	☐
WGS - Clonal Complex in silico	☐	☐	☐	☐
WGS - cgMLST	☐	☐	☐	☐
WGS - wgMLST	☐	☐	☐	☐
WGS - coreSNP analysis	☐	☐	☐	☐
WGS - wgSNP analysis	☐	☐	☐	☐
Antibiotic susceptibility testing	☐	☐	☐	☐
Other	☐	☐	☐	☐

Other (please specify)

24. Are laboratory data stored in databases at the national level?

- ◯ Yes
- ◯ No

25. If yes, which information on isolates is collected

	National level	Sub-national / Regional level	Local level	Intersectorial: human, animal, food	Not shared
Type of specimen	☐	☐	☐	☐	☐
Sampler	☐	☐	☐	☐	☐
Date of sample collection	☐	☐	☐	☐	☐
Place of sample collection	☐	☐	☐	☐	☐
Date of sample receipt	☐	☐	☐	☐	☐
Date of laboratory result	☐	☐	☐	☐	☐
Other	☐	☐	☐	☐	☐

Other (please specify)

26. Please, provide the name and contact details of the National Reference Laboratory in charge of *Listeria* spp. in milk-producing animals.

Institution

City

Website

27. Information on biosecurity measures in place are shared at which level?

- ☐ Only locally
- ☐ Sub-national / Regional level
- ☐ National level
- ☐ Other (please specify)

28. Basing on the biosecurity measures in place, are farms categorized depending the level of risk?

- ◯ Yes
- ◯ No

LISTERIA IN HUMANS AND IN DAIRY PRODUCTS FOOD CHAIN - SECTION B

SURVEILLANCE SYSTEM EVALUATION

29. Has the surveillance system been evaluated?

Please, consider any known evaluations of functioning, performance, organizational aspects, and/or cost-effectiveness.

- ○ Yes
- ○ No

30. Which method of evaluation has been used?

- ☐ Auto-evaluation
- ☐ OASIS method
- ☐ SERVAL method
- ☐ Other (please specify)

31. Please, provide contact details of the institution that conducted the surveillance system evaluation.

Institution

City
