# Supplementary material for: Mapping food surveillance chains through different sectors
Source: Front Public Health. 2023 Apr 18;11:1129851. doi: 10.3389/fpubh.2023.1129851 (PMC10151742; doi:10.3389/fpubh.2023.1129851)
Supplement: Supplementary file 1 [file Data_Sheet_1.ZIP › LISTERIA - SECTION C.pdf]

LISTERIA IN HUMANS AND IN DAIRY PRODUCTS FOOD CHAIN - SECTION C

FOOD CHAIN SURVEILLANCE

Dear participant,
in the context of the OHEJP Matrix, Work-Package 2 (Best-practices and multi-sectorial collaboration) implemented this online questionnaire to collect information about the surveillance of **Listeria** in the **dairy products food chain**, in three sectors: public health, animal health, and food safety.

We would like to thank you for your willingness to fill in the questionnaire for the **food safety** sector.

Please find hereby some information regarding personal data processing.

Under Articles 13 and 14 of Regulation (EU) 2016/679, the personal data processing concerns the personal data - name, family name, institution, email address - of those who answer the questionnaire as part of the Matrix project.
The data controller is the legal representative of Istituto Zooprofilattico Sperimentale Abruzzo e Molise "G. Caporale" – Teramo (Italy), www.izs.it - protocollo@pec.izs.it, +3908613321. The contact details of the DPO of the Institute are: dpo@izs.it, dpo@pec.izs.it, +39 0861 3321.
Personal data collected will be processed for purposes connected with the handling of the contractual requirements related to the management of the Matrix project (art. 6, § 1, letter b) of Regulation).
The personal data provided will not be subject to communication and/or dissemination.
All personal data collected will be processed electronically on digital medium using the specific information systems and, in any case, the processing is made exclusively by personnel in charge. All data collected will be retained anonymously on digital.
At any time, data subjects have the right to ask the data controller for accessing their personal data, confirming such data exist, to know the content, the origin, and the processing terms, to request the update, the rectification, the erasure, the transformation into anonymity or the blocking of the data processed in breach of the law or to object the processing. The related request should be made by contacting the Data Protection Officer or the supervisory authority, in particular in the Member State of his or her habitual residence.

* 1. Country

* 2. Contact info of the person replying to the questionnaire

Name and Surname

Institution

E-mail address

LISTERIA IN HUMANS AND IN DAIRY PRODUCTS FOOD CHAIN - SECTION C

DAIRY PRODUCTION PLANTS & RETAIL

3. Are there any surveillance* activities in place for *Listeria* spp. in **dairy production plants** in your country?

**Surveillance understood as "Targeted Surveillance that is focused on one or more pre-defined hazards (disease, condition, biological, chemical or physical agent, or event) often this form of surveillance uses diagnostic tests for the detection of particular pathogens (e.g. molecular diagnostic methods)." (EJP ORION Glossary)*

- ◯ YES
- ◯ NO

4. Are different types of surveillance activities in place for *Listeria* spp. in dairy products in **dairy production plants** carrying out based on:

	EU legal obligation	National legal obligation	Voluntary based
Official control program	☐	☐	☐
Own check	☐	☐	☐
Baseline survey	☐	☐	☐
Monitoring program	☐	☐	☐
Research project	☐	☐	☐
Outbreak investigation	☐	☐	☐
Other	☐	☐	☐

Other (please specify)

5. Who are the actors in charge of carrying out surveillance activities for *Listeria* spp. in **dairy production plants**?

- ☐ Food business operators
- ☐ Official Control Authorities
- ☐ Other (please specify)

6. How often is sampling performed according to surveillance types in **dairy production plants**?

	Ongoing	Monthly	Quarterly	As required *(E.g. as a part of a defined study or during a defined period)*
Official control program	☐	☐	☐	☐
Own check	☐	☐	☐	☐
Baseline survey	☐	☐	☐	☐
Monitoring program	☐	☐	☐	☐
Research project	☐	☐	☐	☐
Outbreak investigation	☐	☐	☐	☐
Other	☐	☐	☐	☐

Other (please specify)

7. What type of specimens are collected during surveillance activities for *Listeria* spp. in **dairy production plants**?

	Official control program	Own check	Baseline survey	Monitoring program	Research project	Outbreak investigation
Raw milk	☐	☐	☐	☐	☐	☐
Pasteurized milk	☐	☐	☐	☐	☐	☐
Dairy food ready for market	☐	☐	☐	☐	☐	☐
Food contact surfaces	☐	☐	☐	☐	☐	☐
Non-food contact surfaces,	☐	☐	☐	☐	☐	☐
Equipment	☐	☐	☐	☐	☐	☐
Hygienic facilities	☐	☐	☐	☐	☐	☐
Refrigerators	☐	☐	☐	☐	☐	☐
Ripening chambers	☐	☐	☐	☐	☐	☐
Water	☐	☐	☐	☐	☐	☐
Other	☐	☐	☐	☐	☐	☐

Other (please specify)

8. At what time of the dairy production are the specimens collected?

- ☐ Before cleaning and disinfection
- ☐ After cleaning and disinfection
- ☐ Earlier during the day
- ☐ Later during the day
- ☐ Other (please specify)

[   ]

9. In which area of the cheese production plants are the specimens collected?

	Food producing environment	Ingredients or food	Personnel	Cleaning materials
Unloading area - Reception of raw milk and ingredients	☐	☐	☐	☐
Storage of raw milk	☐	☐	☐	☐
Production lines	☐	☐	☐	☐
Cold room	☐	☐	☐	☐
Ripening room	☐	☐	☐	☐
Packaging area	☐	☐	☐	☐
Loading area	☐	☐	☐	☐
Storage area *(packaging materials, detergents, etc.)*	☐	☐	☐	☐
Facilities *(WC, etc.)*	☐	☐	☐	☐
Administrative unit	☐	☐	☐	☐
Other	☐	☐	☐	☐

Other (please specify)

[   ]

10. Do surveillance activities in place for *Listeria* spp. in dairy production plants also include data collection on:

- [ ] Production flow mapping
- [ ] Personnel movement
- [ ] Cleaning and sanitation procedures
- [ ] Critical control points
- [ ] Review of the HACCP plan
- [ ] Verification routines of critical control points and product safety control
- [ ] Review of shelf-life study
- [ ] Other (please specify)

11. Are there any surveillance activities in place for *Listeria* spp. in dairy products at **retail level** in your country?

- ( ) YES
- ( ) NO

12. Are different types of surveillance in place carried out for *Listeria* spp. in dairy products at **retail level** carrying out based on:

	EU legal obligation	National legal obligation	Voluntary based
Official control program	[ ]	[ ]	[ ]
Own check	[ ]	[ ]	[ ]
Baseline survey	[ ]	[ ]	[ ]
Monitoring program	[ ]	[ ]	[ ]
Research project	[ ]	[ ]	[ ]
Outbreak investigation	[ ]	[ ]	[ ]
Other	[ ]	[ ]	[ ]

Other (please specify)

13. Who are the actors in charge of carrying out surveillance activities for *Listeria* spp. in dairy products **at retail level**?

- [ ] Food business operators
- [ ] Official Control Authorities
- [ ] Other (please specify)

14. How often is sampling performed according to surveillance types in dairy products **at retail level**?

	Ongoing	Monthly	Quarterly	As required *(E.g. as a part of a defined study or during a defined period)*
Official control program	☐	☐	☐	☐
Own check	☐	☐	☐	☐
Baseline survey	☐	☐	☐	☐
Monitoring program	☐	☐	☐	☐
Research project	☐	☐	☐	☐
Outbreak investigation	☐	☐	☐	☐
Other	☐	☐	☐	☐

Other (please specify)

15. What information is collected for food products?

- ☐ Compliance with criteria in 2073/2005
- ☐ Concentration of Listeria at the sampling time
- ☐ Concentration of Listeria at the end of shelf life
- ☐ Presence of Listeria
- ☐ Other (please specify)

16. In case of designed surveillance programs, how is the sampling program designed?

- ☐ To investigate the exposure of Listeria in general
- ☐ Targeted to special segments of producers* (e.g small scale producers, unpasteurized milk)
- ☐ Targeted to consumer groups* (e.g vulnerable consumers, high amount consumers of the particular food)
- ☐ Import/export control
- ☐ Other (please specify)

17. * Please specify:

18. Is information about the expiration date of foodstuff sampled collected and reported at national level?

- ◯ Yes
- ◯ No

19. What types of specimens are collected during routine surveillance activities **at retail level**?

- ☐ Food
- ☐ Food handling areas
- ☐ Equipment
- ☐ Personnel
- ☐ Other (please specify)

20. Which metadata are collected during routine surveillance activities?

	Production level	Distribution level	Retail level	Border control by export or import
Registration number of the FBO *(FBO: Food business operator)*	☐	☐	☐	☐
Production date	☐	☐	☐	☐
Expiry date	☐	☐	☐	☐
Batch number	☐	☐	☐	☐
Product size	☐	☐	☐	☐
Animal specie (milk-producing animal)	☐	☐	☐	☐
Storage temperature	☐	☐	☐	☐
Packing conditions	☐	☐	☐	☐
Small scale or large scale FBO	☐	☐	☐	☐
Other	☐	☐	☐	☐

Other (please specify)

LISTERIA IN HUMANS AND IN DAIRY PRODUCTS FOOD CHAIN - SECTION C

DATA MANAGEMENT AND LABORATORY METHODS

21. Are data on surveillance activities in place for *Listeria* spp. in dairy products, at a different stages of the food chain, stored in electronic data collection systems at the national level?

- ○ Yes
- ○ No

22. What kind of information collected during surveillance is shared at the national level?

- ☐ Type of specimen (food, environmental, etc.)
- ☐ Food item
- ☐ Expiration date
- ☐ Sampler
- ☐ Date of sample collection
- ☐ Time of sample collection (before or after disinfection, end of production, etc.)
- ☐ Place of sample collection
- ☐ Stage of processing
- ☐ Sampling context (e.g. monitoring or official control)
- ☐ Other (please specify)

23. Please, provide the name and contact details of the institution in charge of collecting and storing data derived from surveillance activities in place for *Listeria* spp. in dairy products, at different stages of the food chain, at the national level.

Institution

City

Website

24. Please specify the laboratory test used routinely for each type of specimen:

	Food product	Food production environment	Water
PCR	☐	☐	☐
ISO 11290-1 (detection)	☐	☐	☐
ISO 11290-2 (enumeration)	☐	☐	☐
Other culture dependent methods	☐	☐	☐
Culture dependent methods + PCR	☐	☐	☐
Other	☐	☐	☐

Other (please specify)

25. Are the Listeria strains characterized?

- ◯ Yes
- ◯ No

26. If yes, which laboratory methods are used routinely to characterize Listeria strains?

	Always	Sometimes	Never
Serotyping	○	○	○
PCR serotyping	○	○	○
MLST	○	○	○
PFGE	○	○	○
Whole genome sequencing	○	○	○
WGS - PCR serotyping in silico	○	○	○
WGS - MLST in silico	○	○	○
WGS - Clonal Complex in silico	○	○	○
WGS - cgMLST	○	○	○
WGS - wgMLST	○	○	○
WGS - coreSNP analysis	○	○	○
WGS - wgSNP analysis	○	○	○
Antibiotic susceptibility testing	○	○	○
Other	○	○	○

Other (please specify)

27. How do you share the results of laboratory methods? Please check for each diagnostic test one or more possibilities

	National level	Sub-national / Regional level	Local level	Intersectorial: human, animal, food	Not shared
PCR	☐	☐	☐	☐	☐
Culture dependent methods	☐	☐	☐	☐	☐
Culture dependent methods + PCR	☐	☐	☐	☐	☐
Enumeration	☐	☐	☐	☐	☐
Serotyping	☐	☐	☐	☐	☐
PCR serotyping	☐	☐	☐	☐	☐
MLST	☐	☐	☐	☐	☐
PFGE	☐	☐	☐	☐	☐
Whole genome sequencing	☐	☐	☐	☐	☐
WGS - PCR serotyping in silico	☐	☐	☐	☐	☐
WGS - MLST in silico	☐	☐	☐	☐	☐
WGS - Clonal Complex in silico	☐	☐	☐	☐	☐
WGS - cgMLST	☐	☐	☐	☐	☐
WGS - wgMLST	☐	☐	☐	☐	☐
WGS - coreSNP analysis	☐	☐	☐	☐	☐
WGS - wgSNP analysis	☐	☐	☐	☐	☐
Antibiotic susceptibility testing	☐	☐	☐	☐	☐
Other	☐	☐	☐	☐	☐

Other (please specify)

28. When are the results shared for each analytical method used?

	Routinely	Outbreak investigation	Research	As required *(E.g. as a part of a defined study or during a defined period)*
PCR	☐	☐	☐	☐
Culture dependent methods	☐	☐	☐	☐
Culture dependent methods + PCR	☐	☐	☐	☐
Enumeration	☐	☐	☐	☐
Serotyping	☐	☐	☐	☐
PCR serotyping	☐	☐	☐	☐
MLST	☐	☐	☐	☐
PFGE	☐	☐	☐	☐
Whole genome sequencing	☐	☐	☐	☐
WGS - PCR serotyping in silico	☐	☐	☐	☐
WGS - MLST in silico	☐	☐	☐	☐
WGS - Clonal Complex in silico	☐	☐	☐	☐
WGS - cgMLST	☐	☐	☐	☐
WGS - wgMLST	☐	☐	☐	☐
WGS - coreSNP analysis	☐	☐	☐	☐
WGS - wgSNP analysis	☐	☐	☐	☐
Antibiotic susceptibility testing	☐	☐	☐	☐
Other	☐	☐	☐	☐

Other (please specify)

29. Are laboratory data resulting from surveillance activities in place for *Listeria* spp. in dairy products, at different stages of the food chain, stored in databases at the national level?

- ◯ Yes
- ◯ No

30. If yes, which information is collected

	National level	Sub-national / Regional level	Local level	Intersectorial: human, animal, food	Not shared
Type of specimen *(food, environmental, etc.)*	☐	☐	☐	☐	☐
Food item	☐	☐	☐	☐	☐
Expiration date	☐	☐	☐	☐	☐
Sampler	☐	☐	☐	☐	☐
Date of sample collection	☐	☐	☐	☐	☐
Place of sample collection	☐	☐	☐	☐	☐
Stage of processing	☐	☐	☐	☐	☐
Sampling context	☐	☐	☐	☐	☐
Date of sample receipt	☐	☐	☐	☐	☐
Date of laboratory result	☐	☐	☐	☐	☐
Name of laboratory-confirmed pathogen	☐	☐	☐	☐	☐
Other	☐	☐	☐	☐	☐

Other (please specify)

[ ]

31. Please, provide the name and contact details of the National Reference Laboratory in charge of *Listeria* spp. detection, enumeration, and molecular typing in food and in food production environment.

Institution [ ]

City [ ]

Website [ ]

LISTERIA IN HUMANS AND IN DAIRY PRODUCTS FOOD CHAIN - SECTION C

SURVEILLANCE SYSTEM EVALUATION

32. Has the surveillance system been evaluated?

Please, consider any known evaluations of functioning, performance, organizational aspects, and/or cost-effectiveness.

- ◯ Yes
- ◯ No

33. Which method of evaluation has been used?

- ☐ Auto-evaluation
- ☐ OASIS method
- ☐ SERVAL method
- ☐ Other (please specify)

34. Please, provide contact details of the institution that conducted the surveillance system evaluation.

Institution

City
