# Supplementary material for: Mapping food surveillance chains through different sectors
Source: Front Public Health. 2023 Apr 18;11:1129851. doi: 10.3389/fpubh.2023.1129851 (PMC10151742; doi:10.3389/fpubh.2023.1129851)
Supplement: Supplementary file 1 [file Data_Sheet_1.ZIP › SALMONELLA - SECTION A.pdf]

SALMONELLA IN HUMANS AND PORK MEAT FOOD CHAIN - SECTION A

SURVEILLANCE OF DISEASE IN HUMANS

Dear participant,
in the context of the OHEJP Matrix, Work-Package 2 (Best-practices and multi-sectorial collaboration) implemented this online questionnaire to collect information about the surveillance of **Salmonella** in the **pork meat food chain**, in three sectors: public health, animal health, and food safety.

We would like to thank you for your willingness to fill in the questionnaire for the **public health** sector.

Please find hereby some information regarding personal data processing.

Under Articles 13 and 14 of Regulation (EU) 2016/679, the personal data processing concerns the personal data - name, family name, institution, email address - of those who answer the questionnaire as part of the Matrix project.
The data controller is the legal representative of Istituto Zooprofilattico Sperimentale Abruzzo e Molise “G. Caporale” – Teramo (Italy), www.izs.it - protocollo@pec.izs.it, +3908613321. The contact details of the DPO of the Institute are: dpo@izs.it, dpo@pec.izs.it, +39 0861 3321.
Personal data collected will be processed for purposes connected with the handling of the contractual requirements related to the management of the Matrix project (art. 6, § 1, letter b) of Regulation).
The personal data provided will not be subject to communication and/or dissemination.
All personal data collected will be processed electronically on digital medium using the specific information systems and, in any case, the processing is made exclusively by personnel in charge. All data collected will be retained anonymously on digital.
At any time, data subjects have the right to ask the data controller for accessing their personal data, confirming such data exist, to know the content, the origin, and the processing terms, to request the update, the rectification, the erasure, the transformation into anonymity or the blocking of the data processed in breach of the law or to object the processing. The related request should be made by contacting the Data Protection Officer or the supervisory authority, in particular in the Member State of his or her habitual residence.

* 1. Country

* 2. Contact info of the person replying to the questionnaire

Name and Surname

Institution

E-mail address

SALMONELLA IN HUMANS AND PORK MEAT FOOD CHAIN - SECTION A

OFFICIAL SURVEILLANCE

3. Is Salmonellosis a notifiable disease* in your country?

** "A disease that, by law, must be reported to public health authorities upon diagnosis." (EJP ORION Glossary)*

- ◯ YES
- ◯ NO

4. Is there any legal (official) “case definition” and/or “outbreak definition” in your country?

- ◯ YES
- ◯ NO

5. If yes, please specify the case-definition both for “probable” and/or “confirmed case”

[ ]

6. Which is the source of information for the reported data on confirmed human cases?

- ☐ Hospital/clinical Laboratory
- ☐ Local Laboratory
- ☐ Reference Laboratory
- ☐ General Practitioner
- ☐ Hospital physicians
- ☐ Other (please specify)

[ ]

7. Is the data collection for human cases performed for:

	Case based	Aggregated
Probable case	☐	☐
Confirmed case	☐	☐
Outbreak	☐	☐
Other	☐	☐

Other (please specify)

[ ]

8. Please indicate the types of information routinely collected by official surveillance*.

Demographic data:

** Surveillance understood as "Hazard-specific (Targeted) Surveillance: The planned collection of precise data on the presence of a specific disease or pathogen (hazard) within a defined population". (EJP ORION Glossary)*

- ☐ Age (or date of birth)
- ☐ Gender
- ☐ Potential risk factors
  (e.g. transplantation, immunodeficiency etc.)
- ☐ Profession
- ☐ Occupational exposure
- ☐ Place of residence*
- ☐ Travel history
- ☐ Other information (please specify)

9. * Please specify the level of detail (address, city, municipality, etc.)

10. Please indicate the types of information routinely collected by official surveillance.

Epidemiological data:

- ☐ Case status (probable or confirmed)
- ☐ Date of notification
- ☐ Source of notification
- ☐ Probable or confirmed place of exposure* - Restaurant
- ☐ Probable or confirmed place of exposure* - Home
- ☐ Probable or confirmed place of exposure* - Farm
- ☐ Probable or confirmed place of exposure* - Travel related
- ☐ Probable or confirmed type of exposure - Food
- ☐ Probable or confirmed type of exposure - Contact with animals
- ☐ Probable or confirmed type of exposure - Link with other cases
- ☐ Probable or confirmed type of exposure - Occupational exposure
- ☐ Probable or confirmed date of exposure **
- ☐ Other (please specify)

11. * If this information is collected please specify the geographical detail at which information is recorded:

12. ** If this information is collected please specify what kind of information is recorded:

13. Please indicate the types of information routinely collected by official surveillance.

Clinical data:

- ☐ Date of clinical onset
- ☐ Date of recovery*
  (e.g. date of the resolution of symptoms, date of discharge from the hospital, etc.)
- ☐ Fatal (yes / no)
- ☐ Date of death
- ☐ Hospitalized (yes / no)
- ☐ Symptoms
  (e.g.: asymptomatic, fever, diarrhoea, abdominal pain, vomiting, other, unknown)
- ☐ Treatment provided
- ☐ Other (please specify)

14. * If this information is collected please specify:

15. Please indicate the types of information routinely collected by official surveillance.

Laboratory data:

- [ ] Type of specimen
(rectal swab, stool, blood)
- [ ] Sampler
(institution that collects clinical specimen e.g. hospital, local laboratory etc.)
- [ ] Date of sample collection
- [ ] Date of sample receipt
- [ ] Date of laboratory results
- [ ] Laboratory results - Detection
- [ ] Laboratory results - Serology
- [ ] Laboratory results - Characterization
- [ ] Other (please specify)

16. How often are the data collected by official surveillance?

- ○ Ongoing
- ○ Monthly
- ○ Quarterly
- ○ As required
- ○ Other (please specify)

17. If the data collection is “ongoing”, what is the required notification time period?

- ○ 24 hours
- ○ 48 hours
- ○ one week
- ○ two weeks
- ○ Other (please specify)

18. Are data on confirmed human cases coming from the local level transmitted to the national level?

- ○ Yes
- ○ No

19. Are data on confirmed human cases collected and stored in electronic data collection systems at the national level?

- ◯ Yes
- ◯ No

20. Please, provide the name and contact details of the institution in charge of collecting and storing data coming from official surveillance on human cases at the national level.

Institution	
City	
Website	

SALMONELLA IN HUMANS AND PORK MEAT FOOD CHAIN - SECTION A

AD HOC DATA COLLECTION

21. Has been/Is in place any ad hoc data collection* of salmonellosis in humans in your country?
If yes, when were/are the data collected?

**Data collection carried out to supplement information from the surveillance system.*

	On going	Monthly	Quarterly	As required *(E.g. as a part of a defined study or during a defined period)*	No
Outbreak investigation	☐	☐	☐	☐	☐
Clinical study	☐	☐	☐	☐	☐
Epidemiological study	☐	☐	☐	☐	☐
Research study	☐	☐	☐	☐	☐
Hospital data collection system	☐	☐	☐	☐	☐
Other	☐	☐	☐	☐	☐

Other (please specify)

22. What data fields are available in the case of ad hoc data collection?

- ☐ Number of human cases
- ☐ Number of hospitalizations
- ☐ Number of deaths
- ☐ Source identified as probable or confirmed
- ☐ Link with other cases
- ☐ Level of evidence
- ☐ Laboratory results
- ☐ Other (please specify)

SALMONELLA IN HUMANS AND PORK MEAT FOOD CHAIN - SECTION A

LABORATORY BASED SURVEILLANCE

23. Please specify the laboratory test used routinely to diagnose salmonellosis for each type of specimen:

	Rectal swab	Fecal material	Blood
PCR	☐	☐	☐
Culture dependent methods	☐	☐	☐
Culture dependent methods + PCR	☐	☐	☐
Other	☐	☐	☐

Other (please specify)

24. Are the *Salmonella* strains characterized?

- ◯ Yes
- ◯ No

25. If yes, which laboratory methods are used routinely to characterize Salmonella strains?

	Always	Sometimes	Never
PCR	◯	◯	◯
Serotyping	◯	◯	◯
MLVA	◯	◯	◯
PFGE	◯	◯	◯
Whole genome sequencing	◯	◯	◯
WGS - MLST in silico	◯	◯	◯
WGS - cgMLST/wgMLST	◯	◯	◯
WGS - SNP analysis	◯	◯	◯
Antibiotic susceptibility testing	◯	◯	◯
Other	◯	◯	◯

Other (please specify)

26. How do you share the results of laboratory methods? Please check for each diagnostic test one or more possibilities

	National level	Sub-national / Regional level	Local level	Intersectorial: human, animal, food	Not shared
PCR	☐	☐	☐	☐	☐
Culture dependent methods	☐	☐	☐	☐	☐
Culture dependent methods + PCR	☐	☐	☐	☐	☐
Serotyping	☐	☐	☐	☐	☐
MLVA	☐	☐	☐	☐	☐
PFGE	☐	☐	☐	☐	☐
Whole genome sequencing	☐	☐	☐	☐	☐
WGS - MLST in silico	☐	☐	☐	☐	☐
WGS - cgMLST/wgMLST	☐	☐	☐	☐	☐
WGS - SNP analysis	☐	☐	☐	☐	☐
Antibiotic susceptibility testing	☐	☐	☐	☐	☐
Other	☐	☐	☐	☐	☐

Other (please specify)

27. Are data on human isolates from local laboratories shared at:

	National level	Sub-national / Regional level	Local level	Intersectorial: human, animal, food	Not shared
Type of specimen *(rectal swab, stool, blood)*	☐	☐	☐	☐	☐
Sampler *(institution that collect clinical specimen e.g. hospital, local laboratory etc.)*	☐	☐	☐	☐	☐
Date of sample collection	☐	☐	☐	☐	☐
Place of sample collection	☐	☐	☐	☐	☐
Date of sample receipt	☐	☐	☐	☐	☐
Date of laboratory result	☐	☐	☐	☐	☐
Other	☐	☐	☐	☐	☐

Other (please specify)

28. Are laboratory data stored in databases at the national level?

- ◯ Yes
- ◯ No

29. If yes who is in charge to add data into the database?

- ☐ Hospital/clinical Laboratory
- ☐ Local Laboratory
- ☐ Reference Laboratory
- ☐ Other (please specify)

30. Is there any interaction between laboratory and human cases databases at national level?

- ◯ Yes
- ◯ No

31. If yes, when data is updated or available on the database, is there any communication system available to immediately inform the organizations involved?

- ◯ Yes
- ◯ No

If yes, please specify

[ ]

32. Is there any interaction between human cases and food databases (common ID) at the national level?

- ◯ Yes
- ◯ No

33. Please, provide name and contact details of the National Reference Laboratory in charge for Salmonella spp. diagnosis in humans.

Institution [ ]

City [ ]

Website [ ]

SURVEILLANCE SYSTEM EVALUATION

34. Has the surveillance system been evaluated?

Please, consider any known evaluations of functioning, performance, organizational aspects, and/or cost-effectiveness.

- ◯ Yes
- ◯ No

35. Which method of evaluation has been used?

- ☐ Auto-evaluation
- ☐ OASIS method
- ☐ SERVAL method
- ☐ Other (please specify)

36. Please, provide contact details of the institution that conducted the surveillance system evaluation.

Institution

City
