# Supplementary material for: Mapping food surveillance chains through different sectors
Source: Front Public Health. 2023 Apr 18;11:1129851. doi: 10.3389/fpubh.2023.1129851 (PMC10151742; doi:10.3389/fpubh.2023.1129851)
Supplement: Supplementary file 1 [file Data_Sheet_1.ZIP › SALMONELLA - SECTION B.pdf]

SALMONELLA IN HUMANS AND PORK FOOD CHAIN - SECTION B

SURVEILLANCE OF DISEASE IN ANIMALS

Dear participant,
in the context of the OHEJP Matrix, the Work-Package 2 (Best-practices and multi-sectorial collaboration) implemented this online questionnaire to collect information about the surveillance of **Salmonella** in the **pork meat food chain**, in three sectors: public health, animal health, and food safety.

We would like to thank you for your willingness to fill in the questionnaire for the **animal health** sector.

Please find hereby some information regarding personal data processing.

Under Articles 13 and 14 of Regulation (EU) 2016/679, the personal data processing concerns the personal data - name, family name, institution, email address - of those who answer the questionnaire as part of the Matrix project.
The data controller is the legal representative of Istituto Zooprofilattico Sperimentale Abruzzo e Molise "G. Caporale" – Teramo (Italy), www.izs.it - protocollo@pec.izs.it, +3908613321. The contact details of the DPO of the Institute are: dpo@izs.it, dpo@pec.izs.it, +39 0861 3321.
Personal data collected will be processed for purposes connected with the handling of the contractual requirements related to the management of the Matrix project (art. 6, § 1, letter b) of Regulation).
The personal data provided will not be subject to communication and/or dissemination.
All personal data collected will be processed electronically on digital medium using the specific information systems and, in any case, the processing is made exclusively by personnel in charge. All data collected will be retained anonymously on digital.
At any time, data subjects have the right to ask the data controller for accessing their personal data, confirming such data exist, to know the content, the origin, and the processing terms, to request the update, the rectification, the erasure, the transformation into anonymity or the blocking of the data processed in breach of the law or to object the processing. The related request should be made by contacting the Data Protection Officer or the supervisory authority, in particular in the Member State of his or her habitual residence.

* 1. Country

* 2. Contact info of the person replying to the questionnaire

Name and Surname

Institution

E-mail address

SALMONELLA IN HUMANS AND PORK FOOD CHAIN - SECTION B

SURVEILLANCE - General

3. Is Salmonellosis in pigs a notifiable disease* in animals in your country?

** "A disease that, by law, must be reported to public health authorities upon diagnosis." (EJP ORION Glossary)*

- ◯ YES
- ◯ NO

4. Is there any legal (official) “case definition” and/or “outbreak definition” in your country?

- ◯ YES
- ◯ NO

5. If yes, please specify the “case definition” and/or “outbreak definition”

SALMONELLA IN HUMANS AND PORK FOOD CHAIN - SECTION B

SURVEILLANCE - Breeding farms

6. Is the surveillance* for Salmonellosis in pigs in **breeding farms** in place in your country?

**Surveillance understood as "Targeted Surveillance that is focused on one or more pre-defined hazards (disease, condition, biological, chemical or physical agent, or event) often this form of surveillance uses diagnostic tests for the detection of particular pathogens (e.g. molecular diagnostic methods)." (EJP ORION Glossary)*

- ◯ YES
- ◯ NO

7. Are different types of surveillance activities in place for Salmonellosis in pigs in **breeding farms** carrying out based on:

	EU legal obligation	National legal obligation	Voluntary based
Official control program	☐	☐	☐
Baseline survey	☐	☐	☐
Monitoring program	☐	☐	☐
Research project	☐	☐	☐
Outbreak investigation	☐	☐	☐
Other	☐	☐	☐

Other (please specify)

8. Who are the actors in charge of carrying out surveillance activities for Salmonellosis in pigs in **breeding farms**?

- ☐ Farmer
- ☐ Official Control Authorities
- ☐ Vet technician/Private Vet
- ☐ Other (please specify)

9. How often are the samplings performed in pigs in **breeding farms**?

	Ongoing	Monthly	Quarterly	As required *(E.g. as a part of a defined study or during a defined period)*
Official control program	☐	☐	☐	☐
Baseline survey	☐	☐	☐	☐
Monitoring program	☐	☐	☐	☐
Research project	☐	☐	☐	☐
Outbreak investigation	☐	☐	☐	☐
Other	☐	☐	☐	☐

Other (please specify)

10. If the data collection is “ongoing”, what is the required notification time period?

- ◯ 24 hours
- ◯ 48 hours
- ◯ one week
- ◯ two weeks
- ◯ Other (please specify)

11. What types of specimens are collected during surveillance activities in pigs in **breeding farms**?

	Official control program	Baseline survey	Monitoring program	Research project	Outbreak investigation
Water	☐	☐	☐	☐	☐
Feed	☐	☐	☐	☐	☐
Fecal material	☐	☐	☐	☐	☐
Blood	☐	☐	☐	☐	☐
Environmental samples	☐	☐	☐	☐	☐
Other	☐	☐	☐	☐	☐

Other (please specify)

12. Do surveillance activities in place for Salmonellosis in pigs in **breeding farms** also include data collection on:

- [ ] Personnel movement
- [ ] Cleaning and sanitation procedures
- [ ] Other (please specify)

SALMONELLA IN HUMANS AND PORK FOOD CHAIN - SECTION B

SURVEILLANCE - Fattening farms

13. Is the surveillance* for Salmonellosis in pigs in **fattening farms** in place in your country?

**Targeted Surveillance that is focused on one or more pre-defined hazards (disease, condition, biological, chemical or physical agent, or event) often this form of surveillance uses diagnostic tests for the detection of particular pathogens (e.g. molecular diagnostic methods)." (EJP ORION Glossary)*

- ○ YES
- ○ NO

14. Are different types of surveillance activities in place for Salmonellosis in pigs in **fattening farms** carrying out based on:

	EU legal obligation	National legal obligation	Voluntary based
Official control program	☐	☐	☐
Baseline survey	☐	☐	☐
Monitoring program	☐	☐	☐
Research project	☐	☐	☐
Outbreak investigation	☐	☐	☐
Other	☐	☐	☐

Other (please specify)

[ ]

15. Who are the actors in charge of carrying out surveillance activities for Salmonellosis in pigs in **fattening farms**?

- ☐ Farmer
- ☐ Official Control Authorities
- ☐ Vet technician/Private Vet
- ☐ Other (please specify)

[ ]

16. How often are the samplings performed in **fattening farms**?

	Ongoing	Monthly	Quarterly	As required *(E.g. as a part of a defined study or during a defined period)*
Official control program	☐	☐	☐	☐
Baseline survey	☐	☐	☐	☐
Monitoring program	☐	☐	☐	☐
Research project	☐	☐	☐	☐
Outbreak investigation	☐	☐	☐	☐
Other	☐	☐	☐	☐

Other (please specify)

17. If the data collection is “ongoing”, what is the required notification time period?

- ◯ 24 hours
- ◯ 48 hours
- ◯ one week
- ◯ two weeks
- ◯ Other (please specify)

18. What types of specimens are collected during surveillance activities in pigs in **fattening farms**?

	Official control program	Baseline survey	Monitoring program	Research project	Outbreak investigation
Water	☐	☐	☐	☐	☐
Feed	☐	☐	☐	☐	☐
Fecal material	☐	☐	☐	☐	☐
Blood	☐	☐	☐	☐	☐
Environmental samples	☐	☐	☐	☐	☐
Other	☐	☐	☐	☐	☐

Other (please specify)

19. Do surveillance activities in place for Salmonellosis in pigs in **fattening farms** also include data collection on:

- [ ] Personnel movement
- [ ] Cleaning and sanitation procedures
- [ ] Other (please specify)

SALMONELLA IN HUMANS AND PORK FOOD CHAIN - SECTION B

SURVEILLANCE - Other pig farms

20. Is the surveillance* for Salmonellosis in pigs in **other pig farms** in place in your country?

**Targeted Surveillance that is focused on one or more pre-defined hazards (disease, condition, biological, chemical or physical agent, or event) often this form of surveillance uses diagnostic tests for the detection of particular pathogens (e.g. molecular diagnostic methods)." (EJP ORION Glossary)*

- ◯ YES
- ◯ NO

21. Are different types of surveillance activities in place for Salmonellosis in pigs in **other pig farms** carrying out based on:

	EU legal obligation	National legal obligation	Voluntary based
Official control program	☐	☐	☐
Baseline survey	☐	☐	☐
Monitoring program	☐	☐	☐
Research project	☐	☐	☐
Outbreak investigation	☐	☐	☐
Other	☐	☐	☐

Other (please specify)

____

22. Who are the actors in charge of carrying out surveillance activities for Salmonellosis in pigs in **other pig farms**?

- ☐ Farmer
- ☐ Official Control Authorities
- ☐ Vet technician/Private Vet
- ☐ Other (please specify)

____

23. How often are the samplings performed in pigs in **other pig farms**?

	Ongoing	Monthly	Quarterly	As required *(E.g. as a part of a defined study or during a defined period)*
Official control program	☐	☐	☐	☐
Baseline survey	☐	☐	☐	☐
Monitoring program	☐	☐	☐	☐
Research project	☐	☐	☐	☐
Outbreak investigation	☐	☐	☐	☐
Other	☐	☐	☐	☐

Other (please specify)

24. If the data collection is “ongoing”, what is the required notification time period?

- ○ 24 hours
- ○ 48 hours
- ○ one week
- ○ two weeks
- ○ Other (please specify)

25. What types of specimens are collected during surveillance activities in pigs in **other pig farms**?

	Official control program	Baseline survey	Monitoring program	Research project	Outbreak investigation
Water	☐	☐	☐	☐	☐
Feed	☐	☐	☐	☐	☐
Fecal material	☐	☐	☐	☐	☐
Blood	☐	☐	☐	☐	☐
Environmental samples	☐	☐	☐	☐	☐
Other	☐	☐	☐	☐	☐

Other (please specify)

26. Do surveillance activities in place for Salmonellosis in pigs in **other pig farms** also include data collection on:

- [ ] Personnel movement
- [ ] Cleaning and sanitation procedures
- [ ] Other (please specify)

SALMONELLA IN HUMANS AND PORK FOOD CHAIN - SECTION B

SURVEILLANCE - Animal movements

27. Is the surveillance* for Salmonellosis in pigs during **animal movements** in place in your country?

**Targeted Surveillance that is focused on one or more pre-defined hazards (disease, condition, biological, chemical or physical agent, or event) often this form of surveillance uses diagnostic tests for the detection of particular pathogens (e.g. molecular diagnostic methods)." (EJP ORION Glossary)*

- ◯ YES
- ◯ NO

28. Are different types of surveillance activities in place for Salmonellosis in pigs during **animal movements** carrying out based on:

	EU legal obligation	National legal obligation	Voluntary based
Official control program	☐	☐	☐
Baseline survey	☐	☐	☐
Monitoring program	☐	☐	☐
Research project	☐	☐	☐
Outbreak investigation	☐	☐	☐
Other	☐	☐	☐

Other (please specify)

____

29. Who are the actors in charge of carrying out surveillance activities for Salmonellosis in pigs during **animal movements**?

- ☐ Farmer
- ☐ Official Control Authorities
- ☐ Vet technician/Private Vet
- ☐ Transporter
- ☐ Other (please specify)

____

30. How often are the samplings performed in pigs during **animal movements**?

	Ongoing	Monthly	Quarterly	As required *(E.g. as a part of a defined study or during a defined period)*
Official control program	☐	☐	☐	☐
Baseline survey	☐	☐	☐	☐
Monitoring program	☐	☐	☐	☐
Research project	☐	☐	☐	☐
Outbreak investigation	☐	☐	☐	☐
Other	☐	☐	☐	☐

Other (please specify)

31. If the data collection is "ongoing", what is the required notification time period?

- ◯ 24 hours
- ◯ 48 hours
- ◯ one week
- ◯ two weeks
- ◯ Other (please specify)

32. What types of specimens are collected during surveillance activities in pigs during **animal movements**?

	Official control program	Baseline survey	Monitoring program	Research project	Outbreak investigation
Fecal material	☐	☐	☐	☐	☐
Blood	☐	☐	☐	☐	☐
Environmental samples	☐	☐	☐	☐	☐
Other	☐	☐	☐	☐	☐

Other (please specify)

33. Do surveillance activities in place for Salmonellosis in pigs during **animal movements** also include data collection on:

- [ ] Personnel movement
- [ ] Cleaning and sanitation procedures
- [ ] Carrier route
- [ ] Other (please specify)

SALMONELLA IN HUMANS AND PORK FOOD CHAIN - SECTION B

SURVEILLANCE - Increased mortality

34. Is surveillance* for Salmonellosis in pigs in place in case of increased mortality and/or death of suspect animals in **breeding farms**?

**Surveillance understood as "The real-time (or near real-time) collection, analysis, interpretation and dissemination of health-related data to enable the early identification of the impact (or absence of impact) of potential human or veterinary public health threats which require effective public health action". (EJP ORION Glossary)*

- ◯ Yes
- ◯ No

35. In case of yes, which organs/tissues of the animal are investigated?

[ ]

36. Is surveillance for Salmonellosis in pigs in place in case of increased mortality and/or death of suspect animals in **fattening farms**?

- ◯ Yes
- ◯ No

37. In case of yes, which organs/tissues of the animal are investigated?

[ ]

38. Is surveillance for Salmonellosis in pigs in place in case of increased mortality and/or death of suspect animals in **other farms**?

- ◯ Yes
- ◯ No

39. In case of yes, which organs/tissues of the animal are investigated?

[ ]

40. Is surveillance for Salmonellosis in pigs in place in case of increased mortality and/or death of suspect animals during **animal movements**?

- ◯ Yes
- ◯ No

41. In case of yes, which organs/tissues of the animal are investigated?

DATA MANAGEMENT AND LABORATORY METHODS

42. Do you have a National Animal Identification System of pigs in place in your country?

- Yes
- No

43. Do you have a National Animal Movement Identification System of pigs in place in your country?

- Yes
- No

44. Are data on surveillance activities in place for Salmonellosis in pigs, at different stages, stored in electronic data collection systems at the national level?

- Yes
- No

45. Please, provide the name and contact details of the institution in charge of collecting and storing data coming from surveillance activities at the national level.

Institution

City

Website

46. What kind of information collected during surveillance is shared at national level?

- Number of confirmed cases
- Number of suspected cases
- Number of depopulated animals
- Number of dead animals
- Type of specimen *(blood, water, environmental samples, etc.)*
- Sampler
- Date of sample collection
- Place of sample collection
- Sampling context *(official control program, monitoring, etc.)*
- Other (please specify)

47. Please specify the laboratory test used routinely for each type of specimen:

	Fecal material	Blood	Environmental samples	Feed	Water
PCR	☐	☐	☐	☐	☐
ISO 6579-1 (detection)	☐	☐	☐	☐	☐
ISO 6579-1 (enumeration)	☐	☐	☐	☐	☐
Other culture dependent methods	☐	☐	☐	☐	☐
Culture dependent methods + PCR	☐	☐	☐	☐	☐
Other	☐	☐	☐	☐	☐

Other (please specify)

48. Are the Salmonella strains characterized?

◯ Yes

◯ No

49. If yes, which laboratory methods are used routinely to characterize Salmonella strains?

	Always	Sometimes	Never
PCR	◯	◯	◯
Serotyping	◯	◯	◯
MLVA	◯	◯	◯
PFGE	◯	◯	◯
Whole genome sequencing	◯	◯	◯
WGS - MLST in silico	◯	◯	◯
WGS - cgMLST/wgMLST	◯	◯	◯
WGS - SNP analysis	◯	◯	◯
Antibiotic susceptibility testing	◯	◯	◯
Other	◯	◯	◯

Other (please specify)

50. How do you share the results of laboratory methods? Please check for each diagnostic test one or more possibilities:

	National level	Sub-national / Regional level	Local level	Intersectorial: human, animal, food	Not shared
PCR	☐	☐	☐	☐	☐
Culture dependent methods	☐	☐	☐	☐	☐
Culture dependent methods + PCR	☐	☐	☐	☐	☐
Enumeration	☐	☐	☐	☐	☐
Serotyping	☐	☐	☐	☐	☐
MLVA	☐	☐	☐	☐	☐
PFGE	☐	☐	☐	☐	☐
Whole genome sequencing	☐	☐	☐	☐	☐
WGS - MLST in silico	☐	☐	☐	☐	☐
WGS - cgMLST/wgMLST	☐	☐	☐	☐	☐
WGS - SNP analysis	☐	☐	☐	☐	☐
Antibiotic susceptibility testing	☐	☐	☐	☐	☐
Other	☐	☐	☐	☐	☐

Other (please specify)

51. When are the results shared for each analytical method used?

	Routinely	Outbreak investigation	Research	As required *(E.g. as a part of a defined study or during a defined period)*
PCR	☐	☐	☐	☐
Culture dependent methods	☐	☐	☐	☐
Culture dependent methods + PCR	☐	☐	☐	☐
Enumeration	☐	☐	☐	☐
Serotyping	☐	☐	☐	☐
MLVA	☐	☐	☐	☐
PFGE	☐	☐	☐	☐
Whole genome sequencing	☐	☐	☐	☐
WGS - MLST in silico	☐	☐	☐	☐
WGS - cgMLST/wgMLST	☐	☐	☐	☐
WGS - SNP analysis	☐	☐	☐	☐
Antibiotic susceptibility testing	☐	☐	☐	☐
Other	☐	☐	☐	☐

Other (please specify)

[ ]

52. Are laboratory data stored in databases at the national level?

◯ Yes

◯ No

53. If yes, which information on isolates is collected

	National level	Sub-national / Regional level	Local level	Intersectorial: human, animal, food	Not shared
Type of specimen	☐	☐	☐	☐	☐
Sampler	☐	☐	☐	☐	☐
Date of sample collection	☐	☐	☐	☐	☐
Place of sample collection	☐	☐	☐	☐	☐
Date of sample receipt	☐	☐	☐	☐	☐
Date of laboratory result	☐	☐	☐	☐	☐
Other	☐	☐	☐	☐	☐

Other (please specify)

54. Please, provide the name and contact details of the National Reference Laboratory in charge of Salmonella in pigs.

Institution

City

Website

55. Information on biosecurity measures in place are shared at which level?

- ☐ Only locally
- ☐ Sub-national / Regional level
- ☐ National level
- ☐ Other (please specify)

56. Basing on the biosecurity measures in place, are farms categorized depending the level of risk?

- ◯ Yes
- ◯ No

SALMONELLA IN HUMANS AND PORK FOOD CHAIN - SECTION B

SURVEILLANCE SYSTEM EVALUATION

57. Has the surveillance system been evaluated?

Please, consider any known evaluations of functioning, performance, organizational aspects, and/or cost-effectiveness.

- ○ Yes
- ○ No

58. Which method of evaluation has been used?

- ☐ Auto-evaluation
- ☐ OASIS method
- ☐ SERVAL method
- ☐ Other (please specify)

____

59. Please, provide contact details of the institution that conducted the surveillance system evaluation.

Institution ____________________

City ____________________
