# Supplementary material for: Mapping food surveillance chains through different sectors
Source: Front Public Health. 2023 Apr 18;11:1129851. doi: 10.3389/fpubh.2023.1129851 (PMC10151742; doi:10.3389/fpubh.2023.1129851)
Supplement: Supplementary file 1 [file Data_Sheet_1.ZIP › SALMONELLA - SECTION C.pdf]

SALMONELLA IN HUMANS AND IN PORK MEAT FOOD CHAIN - SECTION C

FOOD CHAIN SURVEILLANCE

Dear participant,
in the context of the OHEJP Matrix, Work-Package 2 (Best-practices and multi-sectorial collaboration) implemented this online questionnaire to collect information about the surveillance of **Salmonella** in the **pork meat food chain**, in three sectors: public health, animal health, and food safety.

We would like to thank you for your willingness to fill in the questionnaire for the **food safety** sector.

Please find hereby some information regarding personal data processing.

Under Articles 13 and 14 of Regulation (EU) 2016/679, the personal data processing concerns the personal data - name, family name, institution, email address - of those who answer the questionnaire as part of the Matrix project.
The data controller is the legal representative of Istituto Zooprofilattico Sperimentale Abruzzo e Molise "G. Caporale" – Teramo (Italy), www.izs.it - protocollo@pec.izs.it, +3908613321. The contact details of the DPO of the Institute are: dpo@izs.it, dpo@pec.izs.it, +39 0861 3321.
Personal data collected will be processed for purposes connected with the handling of the contractual requirements related to the management of the Matrix project (art. 6, § 1, letter b) of Regulation).
The personal data provided will not be subject to communication and/or dissemination.
All personal data collected will be processed electronically on digital medium using the specific information systems and, in any case, the processing is made exclusively by personnel in charge. All data collected will be retained anonymously on digital.
At any time, data subjects have the right to ask the data controller for accessing their personal data, confirming such data exist, to know the content, the origin, and the processing terms, to request the update, the rectification, the erasure, the transformation into anonymity or the blocking of the data processed in breach of the law or to object the processing. The related request should be made by contacting the Data Protection Officer or the supervisory authority, in particular in the Member State of his or her habitual residence.

* 1. Country

* 2. Contact info of the person replying to the questionnaire

Name and Surname

Institution

E-mail address

SALMONELLA IN HUMANS AND IN PORK MEAT FOOD CHAIN - SECTION C

SURVEILLANCE - Slaughterhouse

3. Are there any surveillance* activities in place for *Salmonella* spp. in pigs at the **slaughterhouse** in your country?

**Surveillance understood as "Targeted Surveillance that is focused on one or more pre-defined hazards (disease, condition, biological, chemical or physical agent, or event) often this form of surveillance uses diagnostic tests for the detection of particular pathogens (e.g. molecular diagnostic methods)." (EJP ORION Glossary)*

- ◯ YES
- ◯ NO

4. Are different types of surveillance activities in place for *Salmonella* spp. in pigs at the **slaughterhouse** carrying out based on:

	EU legal obligation	National legal obligation	Voluntary based
Official control program	☐	☐	☐
Own check	☐	☐	☐
Baseline survey	☐	☐	☐
Monitoring program	☐	☐	☐
Research project	☐	☐	☐
Outbreak investigation	☐	☐	☐
Other	☐	☐	☐

Other (please specify)

5. Who are the actors in charge of carrying out surveillance activities for *Salmonella* spp. in pigs at the **slaughterhouse**?

- ☐ Slaughter company
- ☐ Official Control Authorities
- ☐ Other (please specify)

6. How often is sampling performed according to surveillance types at the **slaughterhouse**?

	Ongoing	Monthly	Quarterly	As required *(E.g. as a part of a defined study or during a defined period)*
Official control program	☐	☐	☐	☐
Own check	☐	☐	☐	☐
Baseline survey	☐	☐	☐	☐
Monitoring program	☐	☐	☐	☐
Research project	☐	☐	☐	☐
Outbreak investigation	☐	☐	☐	☐
Other	☐	☐	☐	☐

Other (please specify)

7. What types of specimens are collected during surveillance activities for *Salmonella* spp. in pigs at the **slaughterhouse**?

	Official control program	Own check	Baseline survey	Monitoring program	Research project	Outbreak investigation
Carcass swabs	☐	☐	☐	☐	☐	☐
Lymph nodes	☐	☐	☐	☐	☐	☐
Tonsils	☐	☐	☐	☐	☐	☐
Caecal material	☐	☐	☐	☐	☐	☐
Environmental samples	☐	☐	☐	☐	☐	☐
Equipment	☐	☐	☐	☐	☐	☐
Other	☐	☐	☐	☐	☐	☐

Other (please specify)

8. Which is the sample unit of the sample?

	Single	Batch
Carcass swabs	☐	☐
Lymph nodes	☐	☐
Tonsils	☐	☐
Caecal material	☐	☐
Other	☐	☐

Other (please specify)

9. In which slaughter time are the samples collected?

- ☐ Before cleaning and disinfection
- ☐ After cleaning and disinfection
- ☐ Earlier during the day
- ☐ Later during the day
- ☐ Other (please specify)

10. Do surveillance activities in place for *Salmonella* spp. in pigs at the **slaughterhouse** also include data collection on:

- ☐ Production flow mapping
- ☐ Personnel movement
- ☐ Cleaning and sanitation procedures
- ☐ Intervention at slaughter (Scheduled slaughter, logistic slaughter, etc)
- ☐ Decontamination of carcasses (chemical or physical)
- ☐ Other (please specify)

SALMONELLA IN HUMANS AND IN PORK MEAT FOOD CHAIN - SECTION C

SURVEILLANCE - Own consumption

11. Are there any surveillance* activities in place for *Salmonella* spp. in pigs slaughtered for **own consumption** in your country?

**Surveillance understood as "Targeted Surveillance that is focused on one or more pre-defined hazards (disease, condition, biological, chemical or physical agent, or event) often this form of surveillance uses diagnostic tests for the detection of particular pathogens (e.g. molecular diagnostic methods)." (EJP ORION Glossary)*

- ◯ YES
- ◯ NO

12. Are different types of surveillance activities in place for *Salmonella* spp. in pigs slaughtered for **own consumption** carrying out based on:

	EU legal obligation	National legal obligation	Voluntary based
Official control program	☐	☐	☐
Own check	☐	☐	☐
Baseline survey	☐	☐	☐
Monitoring program	☐	☐	☐
Research project	☐	☐	☐
Outbreak investigation	☐	☐	☐
Other	☐	☐	☐

Other (please specify)

13. Who are the actors in charge of carrying out surveillance activities in place for *Salmonella* spp. in pigs slaughtered for **own consumption** in your country?

- ☐ Veterinary Practitioners
- ☐ Official Control Authorities
- ☐ Other (please specify)

14. How often is sampling performed according to surveillance types for **own consumption**?

	Ongoing	Monthly	Quarterly	As required *(E.g. as a part of a defined study or during a defined period)*
Official control program	☐	☐	☐	☐
Own check	☐	☐	☐	☐
Baseline survey	☐	☐	☐	☐
Monitoring program	☐	☐	☐	☐
Research project	☐	☐	☐	☐
Outbreak investigation	☐	☐	☐	☐
Other	☐	☐	☐	☐

Other (please specify)

15. What types of specimens are collected during surveillance activities in place for *Salmonella* spp. in pigs slaughtered for **own consumption**?

	Official control program	Own check	Baseline survey	Monitoring program	Research project	Outbreak investigation
Carcass swabs	☐	☐	☐	☐	☐	☐
Lymph nodes	☐	☐	☐	☐	☐	☐
Tonsils	☐	☐	☐	☐	☐	☐
Caecal material	☐	☐	☐	☐	☐	☐
Environmental samples	☐	☐	☐	☐	☐	☐
Equipment	☐	☐	☐	☐	☐	☐
Other	☐	☐	☐	☐	☐	☐

Other (please specify)

SALMONELLA IN HUMANS AND IN PORK MEAT FOOD CHAIN - SECTION C

SURVEILLANCE - Processing plants

16. Are there any surveillance* activities in place for *Salmonella* spp. in pig meat at the **processing plants** in your country?

**Surveillance understood as "Targeted Surveillance that is focused on one or more pre-defined hazards (disease, condition, biological, chemical or physical agent, or event) often this form of surveillance uses diagnostic tests for the detection of particular pathogens (e.g. molecular diagnostic methods)." (EJP ORION Glossary)*

- ◯ Yes
- ◯ No

17. Are different types of surveillance activities in place for *Salmonella* spp. in pig meat at the **processing plants** carrying out based on:

	EU legal obligation	National legal obligation	Voluntary based
Official control program	☐	☐	☐
Own check	☐	☐	☐
Baseline survey	☐	☐	☐
Monitoring program	☐	☐	☐
Research project	☐	☐	☐
Outbreak investigation	☐	☐	☐
Other	☐	☐	☐

Other (please specify)

____

18. Who are the actors in charge of carrying out surveillance activities for *Salmonella* spp. in pig meat at the **processing plants**?

- ☐ Cutting plants
- ☐ Official Control Authorities
- ☐ Other (please specify)

____

19. How often is sampling performed according to surveillance types at the **processing plants**?

	Ongoing	Monthly	Quarterly	As required *(E.g. as a part of a defined study or during a defined period)*
Official control program	☐	☐	☐	☐
Own check	☐	☐	☐	☐
Baseline survey	☐	☐	☐	☐
Monitoring program	☐	☐	☐	☐
Research project	☐	☐	☐	☐
Outbreak investigation	☐	☐	☐	☐
Other	☐	☐	☐	☐

Other (please specify)

[ ]

20. What types of specimens are collected during surveillance activities at the **processing plants**?

	Official control program	Own check	Baseline survey	Monitoring program	Research project	Outbreak investigation
Fresh meat	☐	☐	☐	☐	☐	☐
Minced meat	☐	☐	☐	☐	☐	☐
Meat preparations	☐	☐	☐	☐	☐	☐
Mechanically separated meat	☐	☐	☐	☐	☐	☐
Environmental samples	☐	☐	☐	☐	☐	☐
Equipment	☐	☐	☐	☐	☐	☐
Other	☐	☐	☐	☐	☐	☐

Other (please specify)

[ ]

21. In which stage of the secondary processing are the samples collected?

	Fresh meat	Minced meat	Meat preparations	Mechanically separated meat	Environmental samples	Equipment
Portioning	☐	☐	☐	☐	☐	☐
Grading and packaging	☐	☐	☐	☐	☐	☐
Secondary chilling (before distribution)	☐	☐	☐	☐	☐	☐
Other	☐	☐	☐	☐	☐	☐

Other (please specify)

SALMONELLA IN HUMANS AND IN PORK MEAT FOOD CHAIN - SECTION C

SURVEILLANCE - Retail

22. Are there any surveillance* activities in place for *Salmonella* spp. in pig meat at **retail level** in your country?

**Surveillance understood as "Targeted Surveillance that is focused on one or more pre-defined hazards (disease, condition, biological, chemical or physical agent, or event) often this form of surveillance uses diagnostic tests for the detection of particular pathogens (e.g. molecular diagnostic methods)." (EJP ORION Glossary)*

◯ YES

◯ NO

23. Are different types of surveillance activities in place for *Salmonella* spp. in pig meat at **retail level** carrying out based on:

	EU legal obligation	National legal obligation	Voluntary based
Official control program	☐	☐	☐
Own check	☐	☐	☐
Baseline survey	☐	☐	☐
Monitoring program	☐	☐	☐
Research project	☐	☐	☐
Outbreak investigation	☐	☐	☐
Other	☐	☐	☐

Other (please specify)

____

24. Who are the actors in charge of carrying out surveillance activities for *Salmonella* spp. in pig meat at **retail level**?

☐ Food business operators

☐ Official Control Authorities

☐ Other (please specify)

____

25. How often is sampling performed according to surveillance types at **retail level**?

	Ongoing	Monthly	Quarterly	As required *(E.g. as a part of a defined study or during a defined period)*
Official control program	☐	☐	☐	☐
Own check	☐	☐	☐	☐
Baseline survey	☐	☐	☐	☐
Monitoring program	☐	☐	☐	☐
Research project	☐	☐	☐	☐
Outbreak investigation	☐	☐	☐	☐
Other	☐	☐	☐	☐

Other (please specify)

26. What types of specimens are collected during routine surveillance activities at **retail level**?

- ☐ Minced meat intended to be eaten raw
- ☐ Meat preparations intended to be eaten raw
- ☐ Minced meat intended to be eaten cooked
- ☐ Meat preparations intended to be eaten cooked
- ☐ Meat products intended to be eaten raw
- ☐ Meat products intended to be eaten cooked
- ☐ Food handling areas
- ☐ Equipment
- ☐ Personnel
- ☐ Other (please specify)

27. In case of designed surveillance programs, how is the sampling program designed?

- ☐ To investigate the exposure to Salmonella spp. in general
- ☐ Targeted to special segments of producers* (e.g small scale producers)
- ☐ Targeted to consumer groups** (e.g vulnerable consumers, high amount consumers of the particular food)
- ☐ Import/export control
- ☐ Other (please specify)

28. * Please specify:

29. ** Please specify:

30. Which metadata are collected during routine surveillance activities?

	Production level	Distribution level	Retail level	Border control by export or import
Registration number of the FBO *(FBO: Food business operator)*	☐	☐	☐	☐
Production date	☐	☐	☐	☐
Expiry date	☐	☐	☐	☐
Batch number	☐	☐	☐	☐
Product size	☐	☐	☐	☐
Storage temperature	☐	☐	☐	☐
Packing conditions	☐	☐	☐	☐
Small scale or large scale FBO	☐	☐	☐	☐
Other	☐	☐	☐	☐

Other (please specify)

SALMONELLA IN HUMANS AND IN PORK MEAT FOOD CHAIN - SECTION C

DATA MANAGEMENT AND LABORATORY METHODS

31. Are data on surveillance activities in place for *Salmonella* spp. in pigs, at different stages of the food chain, stored in electronic data collection systems at the national level?

- ○ Yes
- ○ No

32. What kind of information collected during surveillance is shared at the national level?

- ☐ Type of specimen
  (carcass swab, fresh meat, environmental, etc.)
- ☐ Food item
- ☐ Expiration date
- ☐ Sampler
- ☐ Date of sample collection
- ☐ Time of sample collection
  (before or after disinfection, end of production, etc.)
- ☐ Place of sample collection
- ☐ Stage of processing
- ☐ Sampling context
  (e.g. monitoring or official control)
- ☐ Other (please specify)

33. Please, provide name and contact details of the Institution in charge for collecting and storing data surveillance activities in place for *Salmonella* spp. in pigs, at different stage of the food chain, at national level.

Institution

City

Website

34. Please specify the laboratory test used routinely for each type of specimen:

	Carcass swab	Faeces	Caecal material	Lymph nodes	Tonsils	Mechanically separated meat	Minced meat	Meat preparations	Meat products
PCR	☐	☐	☐	☐	☐	☐	☐	☐	☐
ISO 6579-1 (detection)	☐	☐	☐	☐	☐	☐	☐	☐	☐
ISO 6579-2 (enumeration)	☐	☐	☐	☐	☐	☐	☐	☐	☐
Other culture dependent methods	☐	☐	☐	☐	☐	☐	☐	☐	☐
Culture dependent methods + PCR	☐	☐	☐	☐	☐	☐	☐	☐	☐
Other	☐	☐	☐	☐	☐	☐	☐	☐	☐

Other (please specify)

35. Are the *Salmonella* strains usually genotyped?

- ○ Yes
- ○ No

36. If yes, which laboratory methods are used routinely to characterize *Salmonella* strains?

	Always	Sometimes	Never
PCR	○	○	○
Serotyping	○	○	○
MLVA	○	○	○
PFGE	○	○	○
Whole genome sequencing	○	○	○
WGS - MLST in silico	○	○	○
WGS - cgMLST/wgMLST	○	○	○
WGS - SNP analysis	○	○	○
Antibiotic susceptibility testing	○	○	○
Other	○	○	○

Other (please specify)

37. How do you share the results of laboratory methods? Please check for each diagnostic test one or more possibilities

	National level	Sub-national / Regional level	Local level	Intersectorial: human, animal, food	Not shared
PCR	☐	☐	☐	☐	☐
Culture dependent methods	☐	☐	☐	☐	☐
Culture dependent methods + PCR	☐	☐	☐	☐	☐
Enumeration	☐	☐	☐	☐	☐
Serotyping	☐	☐	☐	☐	☐
MLVA	☐	☐	☐	☐	☐
PFGE	☐	☐	☐	☐	☐
Whole genome sequencing	☐	☐	☐	☐	☐
WGS - MLST in silico	☐	☐	☐	☐	☐
WGS - cgMLST/wgMLST	☐	☐	☐	☐	☐
WGS - SNP analysis	☐	☐	☐	☐	☐
Antibiotic susceptibility testing	☐	☐	☐	☐	☐
Other	☐	☐	☐	☐	☐

Other (please specify)

38. When are the results shared for each analytical method used?

	Routinely	Outbreak investigation	Research	As required *(E.g. as a part of a defined study or during a defined period)*
PCR	☐	☐	☐	☐
Culture dependent methods	☐	☐	☐	☐
Culture dependent methods + PCR	☐	☐	☐	☐
Enumeration	☐	☐	☐	☐
Serotyping	☐	☐	☐	☐
MLVA	☐	☐	☐	☐
PFGE	☐	☐	☐	☐
Whole genome sequencing	☐	☐	☐	☐
WGS - MLST in silico	☐	☐	☐	☐
WGS - cgMLST/wgMLST	☐	☐	☐	☐
WGS - SNP analysis	☐	☐	☐	☐
Antibiotic susceptibility testing	☐	☐	☐	☐
Other	☐	☐	☐	☐

Other (please specify)

39. Are laboratory data resulting from surveillance activities in place for Salmonella in pigs, at different stages of the food chain, stored in databases at national level?

- ◯ Yes
- ◯ No

40. If yes, which information is collected

	National level	Sub-national / Regional level	Local level	Intersectorial: human, animal, food	Not shared
Type of specimen *(rectal swab, meat, environmental, etc.)*	☐	☐	☐	☐	☐
Food item	☐	☐	☐	☐	☐
Expiration date	☐	☐	☐	☐	☐
Sampler	☐	☐	☐	☐	☐
Date of sample collection	☐	☐	☐	☐	☐
Place of sample collection	☐	☐	☐	☐	☐
Stage of processing	☐	☐	☐	☐	☐
Sampling context	☐	☐	☐	☐	☐
Date of sample receipt	☐	☐	☐	☐	☐
Date of laboratory result	☐	☐	☐	☐	☐
Name of laboratory-confirmed pathogen	☐	☐	☐	☐	☐
Other	☐	☐	☐	☐	☐

Other (please specify)

____

41. Please, provide the name and contact details of the National Reference Laboratory in charge of *Salmonella* spp. detection, enumeration, and molecular typing in food and in samples at slaughter.

Institution ____________________

City ____________________

Website ____________________

SALMONELLA IN HUMANS AND IN PORK MEAT FOOD CHAIN - SECTION C

SURVEILLANCE SYSTEM EVALUATION

42. Has the surveillance system been evaluated?

Please, consider any known evaluations of functioning, performance, organizational aspects, and/or cost-effectiveness.

- ○ Yes
- ○ No

43. Which method of evaluation has been used?

- ☐ Auto-evaluation
- ☐ OASIS method
- ☐ SERVAL method
- ☐ Other (please specify)

44. Please, provide contact details of the institution that conducted the surveillance system evaluation.

Institution

City
